# The Role of Hydrophobic Nodes in the Dynamics of Class A β-Lactamases

**DOI:** 10.3389/fmicb.2021.720991

**Published:** 2021-09-21

**Authors:** Edgar Olehnovics, Junqi Yin, Adrià Pérez, Gianni De Fabritiis, Robert A. Bonomo, Debsindhu Bhowmik, Shozeb Haider

**Affiliations:** ^1^Pharmaceutical and Biological Chemistry, University College London School of Pharmacy, London, United Kingdom; ^2^Oak Ridge National Laboratory, National Center for Computational Sciences, Oak Ridge, TN, United States; ^3^Computational Science Laboratory, Barcelona Biomedical Research Park, Universitat Pompeu Fabra, Barcelona, Spain; ^4^Institució Catalana de Recerca i Estudis Avançats, Barcelona, Spain; ^5^Department of Molecular Biology and Microbiology, Case Western Reserve University, Cleveland, OH, United States; ^6^Department of Medicine, School of Medicine, Case Western Reserve University, Cleveland, OH, United States; ^7^Department of Biochemistry, Case Western Reserve University, Cleveland, OH, United States; ^8^Department of Pharmacology, Case Western Reserve University, Cleveland, OH, United States; ^9^Department of Proteomics and Bioinformatics, Case Western Reserve University, Cleveland, OH, United States; ^10^CWRU-Cleveland VAMC Center for Antimicrobial Resistance and Epidemiology (Case VA CARES), Cleveland, OH, United States; ^11^Veterans Affairs Northeast Ohio Healthcare System, Research Service, Cleveland, OH, United States; ^12^Computer Sciences and Engineering Division, Oak Ridge National Laboratory, Oak Ridge, TN, United States

**Keywords:** β-lactamase, class A, hydrophobic nodes, Markov state model, deep learning, molecular dynamics

## Abstract

Class A β-lactamases are known for being able to rapidly gain broad spectrum catalytic efficiency against most β-lactamase inhibitor combinations as a result of elusively minor point mutations. The evolution in class A β-lactamases occurs through optimisation of their dynamic phenotypes at different timescales. At long-timescales, certain conformations are more catalytically permissive than others while at the short timescales, fine-grained optimisation of free energy barriers can improve efficiency in ligand processing by the active site. Free energy barriers, which define all coordinated movements, depend on the flexibility of the secondary structural elements. The most highly conserved residues in class A β-lactamases are hydrophobic nodes that stabilize the core. To assess how the stable hydrophobic core is linked to the structural dynamics of the active site, we carried out adaptively sampled molecular dynamics (MD) simulations in four representative class A β-lactamases (KPC-2, SME-1, TEM-1, and SHV-1). Using Markov State Models (MSM) and unsupervised deep learning, we show that the dynamics of the hydrophobic nodes is used as a metastable relay of kinetic information within the core and is coupled with the catalytically permissive conformation of the active site environment. Our results collectively demonstrate that the class A enzymes described here, share several important dynamic similarities and the hydrophobic nodes comprise of an informative set of dynamic variables in representative class A β-lactamases.

## Introduction

β-lactams are the most frequently prescribed antibacterial drugs due to their minimal toxicity profiles (Bush and Bradford, 2016). They include the derivatives of penicillins, cephalosporins, carbapenems, and monobactams and have in common the presence of a β-lactam ring, which when hydrolysed by nucleophilic serine of target penicillin-binding protein (PBP), leads to irreversible PBP acylation that prevents formation of peptidoglycan transpeptide crosslinks (Tooke et al., 2019). The accumulation of long-lived acyl-enzyme PBP adducts inhibits reproduction of Gram positive and negative bacteria by preventing biosynthesis of new bacterial cell wall layers (Fisher and Mobashery, 2009).

Among a handful of mechanisms, which the bacteria have evolved to survive and grow in the presence of β-lactams, the most problematic phenotypes are observed in Gram-negative bacteria, including *Escherichia coli, Klebsiella pneumoniae*, and *Pseudomonas aeruginosa* (Tooke et al., 2019). These organisms rapidly exchange plasmids that often carry genes encoding broad-spectrum β-lactamase enzymes, found on transposable elements (Davies and Davies, 2010). These enzymes hydrolyse the endocyclic amide bond of the β-lactam ring, releasing the inactivated product in which the β-lactam ring is open and cannot inhibit PBPs (Palzkill, 2018; Supplementary Figure S1). Moreover, there is nothing to prevent these bacteria from simultaneously expressing multiple different β-lactamases received from a single plasmid, especially where selective pressures remain significant (e.g., in a healthcare setting; Fisher and Mobashery, 2016; Partridge et al., 2018).

In most diagnosed cases of Gram-negative infection, class A β-lactamases such as TEM and SHV are often implicated in multidrug resistant phenotype in response to aminopenicillins and early-generation cephalosporins (Tooke et al., 2019). It is also common for the early variants of these enzymes (TEM-1 and SHV-1) to acquire elusively minor point mutations, granting them the extended-spectrum β-lactamase (ESBL) phenotype (as, e.g., in TEM-3 and SHV-2), which extends their catalytic efficiency to include oxyimino-cephalosporins and monobactams (Hart et al., 2016; Bush, 2018).

The rapid gain of function in ESBLs is not only scientifically interesting, but clinically important, because unless a patient is specifically tested positive for ESBL; penicillins and cephalosporins remain the most frequently prescribed antibiotics (Dolk et al., 2018). Only if and when ESBL mediated-resistance is suspected, carbapenems have been used as an effective monotherapy (Harris et al., 2018). In TEM and SHV variants, carbapenems remain effective by forming a stable long-lived acyl-enzyme adduct with the active site serine residue (S70). However, another Class A β-lactamase, KPC-1, can rapidly hydrolyse and deacylate the acyl-enzyme intermediate; a rapid process for which the active site topology in KPC, and in the closely related enzymes like SME, is more energetically favourable (Zafaralla and Mobashery, 1992; Ke et al., 2007; Kalp and Carey, 2008; Fonseca et al., 2012; Chudyk et al., 2014). It was soon found that KPC-2 is identical to KPC-1, and since then many more variants have been discovered, differing by only one or two amino acid substitutions (Arnold et al., 2011). By now, these highly evolved enzymes can be found worldwide and can hydrolyse all clinically available β-lactams, including clavulanates, extended-spectrum cephalosporins, monobactams, and carbapenems (Leavitt et al., 2007; Drawz and Bonomo, 2010; Stoesser et al., 2017; Bush, 2018; Tooke et al., 2020). For example, KPC-2 and SME-1 can both hydrolyse imipenem at 125- and 183-fold higher kcat/Km efficiency, respectively, compared to TEM-1 (Ke et al., 2007). In turn, Gram-negative infections involving these two enzymes have been linked with >40% mortally rates (Cho et al., 2018).

Currently, there exist only four clinically approved treatments against carbapenem-resistant Enterobacterales: avibactam (a mechanism based competitive inhibitor based on a bicyclic-core scaffold) in a combination with advanced generation cephalosporin (ceftazidime); a combination of vaborbactam (monocyclic boronate slowly reversible inhibitor) with a carbapenem (meropenem); and a combination of ceftolozane-tazobactam and a recently approved combination of imipenem-relebactam (Lagacé-Wiens et al., 2014; Cho et al., 2018; Krajnc et al., 2019; Papp-Wallace et al., 2020; Pemberton et al., 2020; Heo, 2021). Since Avibactam was introduced in 2015, naturally occurring mutations in KPC family were shortly reported in North America; conferring significant resistance to ceftazadime-avibactam, albeit with a partially restored susceptibility to carbapenems (Haidar et al., 2017; Shields et al., 2017). Nonetheless, the global dissemination of KPC-2, as well as ESBL variants of TEM and SHV is still an on going concern, placing this whole class A family of enzymes under spotlight.

Although the novel bicyclic-boronate derivatives, including taniborbactam (which is currently undergoing phase III trials), are predicted to be highly effective in the short term, there is no certainty that resistance to these novel active site inhibitors will not emerge after several years in circulation. To work towards addressing such a risk, which history is teaching us to anticipate, this study provides supporting evidence in line with the alternative allosteric approach for inhibition of class A enzymes; a strategy which has already been receiving steady interest in TEM-1 and KPC-2 (Horn and Shoichet, 2004; Meneksedag et al., 2013; Bowman et al., 2015; Avcı et al., 2016; Hart et al., 2016; Grigorenko et al., 2017; Galdadas et al., 2018, 2021).

**Figure 1:**
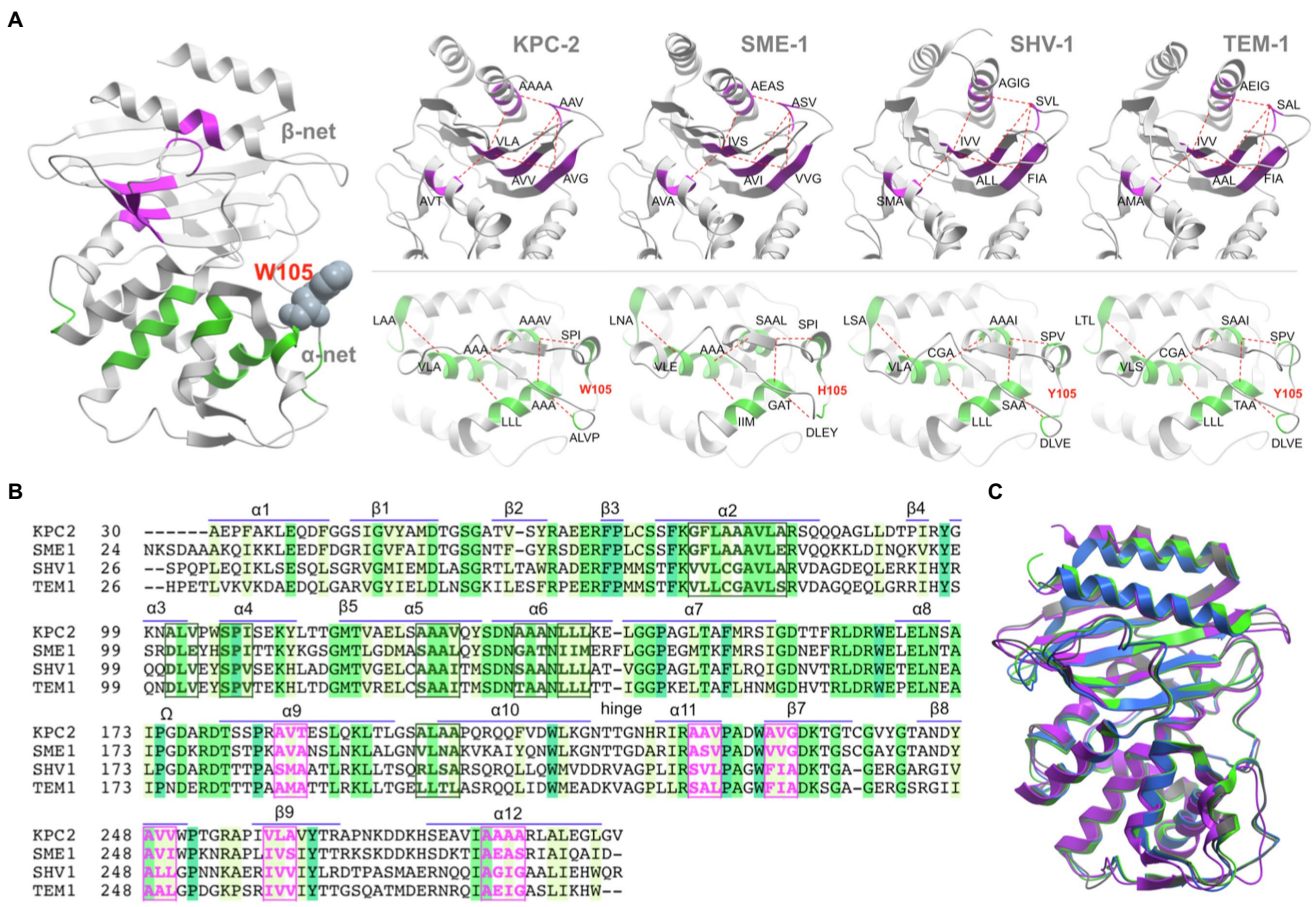
Hydrophobic networks in class A β-lactamases. **(A)** General structure of class A β-lactamase, as represented by the KPC-2 enzyme (PDB id 3DW0). The conservation of α-network (green) and β-network (magenta) has been highlighted for KPC-2 (PDB id 2OV5), SME-1 (PDB id 1DY6), SHV-1 (PDB id 3N4I), and TEM-1 (PDB id 1XPB). **(B)**. Sequence alignment between the four class A β-lactamase sequences. The sequence is annotated by the secondary structure elements. The position of residues in α-network (green box) and β-network (magenta box) is highlighted in bold font. **(C)** In spite of the sequence identity between the enzymes (KPC-2 – grey; SME-1 – blue; SHV-1 – magenta; and TEM-1 – green) is ~35%, the structures can align with each other with <0.91Å rmsd (details in Supplementary Figure S2).

A set of highly conserved stretches of 3–9 hydrophobic residues (each stretch is referred to as a node) has been identified within the core of all class A β-lactamases (Galdadas et al., 2018). The nodes appear as repeats throughout the sequence, both in direct proximity to the active site, stabilising helices α2, α5, and α6 and as flanking residues to those that are directly involved in catalysis (S70, K73, S130, E166, and K234) and active site integrity (N132, T237). Hydrophobic nodes are found to be in local contact with each other (packing), flexibly stabilising the tertiary structure of class A enzymes. The network of hydrophobic interactions was named according to the sub-domains, which they stabilise (α-network or β-network; Galdadas et al., 2018). Both networks contain six nodes each, comprising of 29 and 19 hydrophobic residues, respectively. The low sequence variation of the nodes in the α-domain (α-network) suggests a more conserved functional significance (Figure 1).

From a structural point of view, the hydrophobic core in class A β-lactamases is highly conserved, and therefore the least likely to mutate. This makes it an attractive target for therapeutic intervention. The motivation behind targeting class A β-lactamases *via* an allosteric approach is to inhibit functionally permissive protein conformations by preventing concerted motions, which are involved during substrate processing (functional dynamics) or by altering the kinetics of the enzyme towards catalytically unfavourable configurations or by kinetically biasing the dynamics towards free energy (FE) minima where the active site environment is least able to support the key reaction steps (Laskowski et al., 2009; Motlagh et al., 2014). Mutagenesis studies directed at the hydrophobic network have already shown significant results in KPC-2 enzyme, both experimentally and using molecular dynamics (MD) simulations (Galdadas et al., 2018). Furthermore, the observation that the allosteric signals propagate through the hydrophobic core and reach common structural elements surrounding the active site, despite starting from opposite ends of the protein, in TEM-1 and KPC-2 (Galdadas et al., 2021), warranted a closer look at the dynamic role of hydrophobic network in representative class A β-lactamases. To sample the conformational FE landscape explored by the hydrophobic networks, we performed adaptive sampling equilibrium MD simulations of four representative class A β-lactamases (KPC-2, SME-1, TEM-1, and SHV-1) and investigated the metastability of loops and the hydrophobic nodes *via* Markov State Models (MSM). To visualize the major metastable conformations of hydrophobic network alone, unsupervised low dimensional embeddings were created using a convolutional variational autoencoder. These and various supplementary observations, align well with our previous experimental findings where the highly conserved hydrophobic nodes comprise of an informative set of dynamic variables in all class A β-lactamases.

## Materials and Methods

### Markov State Model-Based Adaptive Sampling Molecular Dynamics Simulations

The crystal structures of KPC-2 (PDB id 2OV5), SHV-1 (PDB id 3N4I), and -SME-1 (PDB id 1DY6)-and-TEM-1 (PDB id 1XPB) were downloaded from the Protein-Data-Bank. From each file, chain A was saved and protonated at pH 7.0 using propka as implemented in the playmolecule (Martínez-Rosell et al., 2017). One intramolecular covalent disulphide bond was specified in each system and structures were hydrated with TIP3P water molecules in a cubic periodic box. Spacing between the protein and the edges of the box was set to 10Å. Sodium or chloride ions were added to neutralise the net charge in each system. The Amber ff14SB force field was used to parameterise protein atoms (Maier et al., 2015); with electrostatic interaction distances set to ≤8Å. Long-range electrostatic interactions were computed using particle mesh Ewald summation method (Wells and Chaffee, 2015). Systems were energy minimised for 1,000 iterations of steepest descent and then equilibrated for 5ns at 1 atmospheric pressure using Berendsen barostat (Feenstra et al., 1999). Initial velocities within each simulation were sampled from Boltzmann distribution at temperature of 300K. Multiple short MSM-based adaptively sampled simulations were run for 60ns in each system using the ACEMD molecular dynamics engine (Harvey et al., 2009; Doerr et al., 2016). Isothermic-isobaric NVT ensemble using a Langevin thermostat with a damping of 0.1ps^−1^ and hydrogen mass repartitioning scheme to achieve time steps of 4fs. Production trajectory frames were saved every 0.1ns. Resulting trajectories are summarised in Table 1.

**Table 1:** Summary of multiple adaptively sampled trajectories. Each trajectory is 60ns (600 frames) at a time step of 0.1ns.

System	Number of trajectories	Total simulation time (μs)
KPC-2	936	56.16
SME-1	268	16.08
TEM-1	404	24.24
SHV-1	593	35.58

### Markov State Models

Pyemma v2.5.7 was used to build the MSM (Scherer et al., 2015). Backbone dihedral angles (φ and ψ) of all residues, and the χ1 angle from the residues of the hydrophobic nodes were chosen as input features. A detailed list of hydrophobic nodes is presented in the supplementary section (Supplementary Table S1). The featurised trajectories were projected onto top three principal components, and then clustered using *k*-means. The optimal number of *k*-means clusters was set to 150. A lag time of 5ns was selected from the implied timescales plot. The MSM was deemed acceptable after passing the Chapman-Kolmogorov (CK) test within narrow confidence intervals. This implies that the model agrees with the data and is therefore statistically significant for use in subsequent steps. To gain access to these confidence intervals, Pyemma’s Bayesian MSM was used to make the final model in each system. Finally, the Transition Path Theory function was used to calculate net flux pathways between the macrostates, originating from state 1. State 1 was chosen as the source because it presented the lowest stationary probability in each system, which make state 1 a reasonable starting point to explain all of the relevant kinetic transitions thought the full FE landscape, i.e., before the net flux eventually reaches the global FE minimum (the sink). The structural results were collected from each perron cluster cluster analysis (PCCA) distribution. These structures represent large-scale variations in protein conformation, which are unique to each given prominent FE minimum.

Fpocket software was used to find pockets in each PCCA frame (Le Guilloux et al., 2009). Vertices generated by fpocket were processed to link the information collected about each pocket to the relevant structural locations where pocket-forming propensities are the highest (cryptic sites). Volumes of these cavities, including the active site volume, sampled within the five or six largest FE minima, are presented in this study.

### Convolutional Variational Autoencoder

Distance maps were built as a function of Cα trajectories of the hydrophobic node residues. Spatial positions of the 48 residues are defined in all class A β-lactamases (Galdadas et al., 2018; Supplementary Table S1). Every fifth frame from the trajectories summarised in Table 1 was saved to build the distance maps. In each of these frames pairwise distances between the relevant Cα atoms, which are ≤8Å were saved as non-zero elements. The resulting distance matrices were stacked in a 3D array. Identical procedure was followed to for all systems. Prior to training, the ordering of the frames in these arrays was randomised, and a simple training vs. validation split of 80:20 was then defined. In Convolutional Variational Autoencoder (CVAE) approach where the training objective was to cluster the conformations of hydrophobic nodes between all four enzymes, the four arrays were concatenated prior to randomisation. During training, a batch-size of 300 was used, and data were re-shuffled after each completed epoch. After completion of the training, signified by converged gradient descent, the complete dataset was embedded for visualisation. To label the embeddings, collective variables (CVs) 1–3, as well as Cα root mean squared deviation (Cα-RMSD) of the hydrophobic node residues were pre-computed for each trajectory frame. CV1, CV2, and CV3 represent the distances between the following pairs of residue’s atoms, respectively: ||105 (Cγ)-167 (Cγ)||_2_, ||105(Cγ)-216(Cβ)||_2_, and ||167(Cγ)-216(Cβ)||_2_. Collectively, these distances are arranged in a triangle and describe the dynamics in the three loops surrounding the active site (α3-α4 loop, the hinge region, and the start of α8 helix), with specific attention to residue 105-side chain conformations. The choice of this CVs is adopted from Galdadas et al. (2018), where analogous distances between these side chains were used in KPC-2.

Illustrations of the CVAE neutral network architecture, which were used, as well as further details about the hyper-parameter choices, are addressed in Supplementary Figure S3. Analogous to CVAE architectures, the objective was to minimise the combined loss (Kingma and Welling, 2014). The Python code for the model implemented in the current work was adopted from Bhowmik et al. (2018) and has been successfully implemented previously (Bhowmik et al., 2018; Romero et al., 2019; Akere et al., 2020; Cho et al., 2021). The combined loss was minimised by gradient descent using RMSprop optimiser, and no dropout was used. The upgraded weights, as well as the training and validation combined losses were saved after every epoch. The two individual losses were monitored during training for signs of overfitting. In the case of VAE, overfitting behaviour often involves the rise of the VAE-loss after the likelihood loss has reached its natural limit (under the conditions of being regularised). Training was stopped as soon as this was observed.

### Structural Analysis

The structural analysis was carried out using Gromacs tools (Abraham et al., 2015), *pytraj* (Roe and Cheatham, 2013) and *mdtraj* (McGibbon et al., 2015). All trajectories were least squares fit to their corresponding crystal structures using Moleculekit as implemented in HTMD tools (Doerr et al., 2016). To analyse relative mobility at different region of the backbone, the MDLovofit algorithm was used (Martínez, 2015). The Cα-RMSD cut off was set as <1Å for the alignment subset. There are three kinds of Root mean square fluctuation (RMSF) plots shown in this study: (a) conventional Cα RMSF plots generated by using all frames in the trajectory; (b) conformational drift plots generated from the MSM-derived PCCA structures relative to the crystal structure; and (c) filtered Cα RMSF plots that highlight backbone regions undergoing the slowest orthogonal linear autocorrelations, where the RMS distances were computed relative to the globally average structure and not the crystal structure. The conformational drift plots in MSM results also include Kruskal Wallis ANOVA values of *p* (Daniel, 1990). These were computed using *scipy.stats.kruskal* function. For covariance overlaps and linear discriminant analysis (LDA), structurally analogous Cα atoms in all four systems were least squares fit relative to the average Cα conformation in KPC-2 using *mdtraj*. Small number of non-homologous insertions and terminal residues were omitted from this calculation to align structurally homologous regions accurately. A further step was added by computing the subspace overlap from vectorised contact maps, which was expected to represent a more robust comparison.

To approximate relative dynamic information of residues within the global dynamics of the systems, normalised mutual information was computed using: [(H(X)+H(Y))/H(X,Y)]−1; where H(X,Y) in a joint entropy and H(X) and H(Y) are marginal entropies of two random variables X and Y. This symmetric function outputs a scalar value between 0 and 1, which represents magnitude of correlation between the two input variables. In this study, {X, Y} were all possible pairs of backbone dihedral angles. Probability densities were estimated using standard histogram method using a small number of 10 bins for each dihedral angle, consistently in all four enzymes.

The trajectories were visualised in Pymol-mdanalysis^1^ and VMD (Humphrey et al., 1996). The structural figures were generated in VMD (Humphrey et al., 1996) and Protein Imager (Tomasello et al., 2020).

## Results and Discussion

### Evolutionary Trends in Structure and Dynamics

KPC-2, SHV-1, SME-1, and TEM-1 β-lactamases are homologs. The average root mean-squared deviation (RMSD) between analogous Cα atoms ranges between 0.38 and 0.90Å (Supplementary Figures S2A–C). Phylogenetic analysis indicates KPC-2 and SME-1 are more similar than TEM-1 and SHV-1 (Supplementary Figure S2D). The sequence identity ranges between 34 and 67% and sequence similarity is between 54 and 81% (Supplementary Figures S2E,F).

The dynamic mobility of the systems was assessed using Cα-RMSD profiles. The low RMSD of the systems are consistent with the previous observation that class A β-lactamases are stable structures when studied on long timescales (Galdadas et al., 2018, 2021; Gobeil et al., 2019). Conventional RMSD methods are unable to differentiate between regions of high vs. low mobility. To resolve this, a fraction (%) of the Cα atoms were used for alignment. Beyond this fraction, there is a steep increase in RMSD value for the rest of the Cα atoms (Supplementary Figure S4). In KPC-2, only 4% of Cα RMSD is greater than 1Å. These residues include the distal flap, the Ω-loop, α7-α8 loop, and the β9-α12 loop. In SME-1, 8% of Cα RMSD is greater than 1Å. The dynamic regions included the α3 helix, hinge-α11 region, and the loop between β8 and β9 strands. In TEM-1, 10% residues displayed Cα RMSD >1Å. These included the α7-α8 loop, the hinge-α11 region, and β8-β9 loop. About 35% of residues showed Cα RMSD>1Å in SHV-1. The regions of high mobility in SHV-1 include the loops between α3-α4, α8-Ω, β7-β8, β8-β9, β9-α12, and the α11-hinge region. The apparent rigidity of these structures is consistent with the experimental data, based on thermal melting experiments, that KPC-2 is a more stable enzyme than many other class A β-lactamases (Mehta et al., 2015).

The structural flexibility was further assessed using Cα RMSF (Supplementary Figure S5). Two different methods were used to assess RMSF. The first method involved filtering all simulated trajectories seven times through separate linear matrices {A_1_,…,A_i_,…,A_7_} to make each RMSF curve. Each matrix (A_i_) was an outer product between one set of left and right time-lagged independent component (IC), scaled by the corresponding i’th largest eigenvalue. These RMSF plots highlight how different regions in backbone can have dynamic correlations at different timescales (Supplementary Figure S5A). Second, was the conventional method of assessing RMSF, over all simulated conformations (Supplementary Figure S5B). Higher RMSF values indicate regions of flexibility. The overall pattern of flexibility is similar in all systems. The largest variance is observed in loop regions that connect secondary structural elements.

The dynamic subspace overlap was calculated from trajectories of structurally analogous Cα atoms, using both raw coordinates and vectorised contact maps with a cut-off distance of <8Å (Supplementary Figure S2G). This was repeated on a subset of Cα atoms from residues of the hydrophobic nodes (Supplementary Figure S2H). The dynamic comparisons based on the contact maps align closely with the anticipated evolutionary trends, as seen in the phylogenetic tree of the four enzymes and their residue similarities. Moreover, the average level of similarity between pairwise dynamics in the four enzymes is greater when comparing the hydrophobic nodes in isolation, indicating that dynamics within the hydrophobic networks are more conserved. Interestingly, the high similarity value observed between dynamics of Cα atoms in TEM-1 and SME-1 (Supplementary Figure S2H, bottom triangle), was absent when repeating a similar procedure using the contact maps as features (Supplementary Figure S2H; top triangle), while all the other comparisons remained relatively consistent.

### Markov State Models

It was possible to build a converged MSM using the backbone dihedral angles of the residues in the hydrophobic nodes for TEM-1 and SHV-1, but not for SME-1 and KPC-2. For better resolution of metastability, MSMs were instead built based on the full set of backbone dihedral angles of all residues and the χ1 angle from the residues in the hydrophobic nodes at a lag time of 5ns. This choice of features allows us to make a valid link between the FE landscape (dominated by dynamics of the loops) in relation to any significant structural differences seen in the hydrophobic node residues.

**Figure 2:**
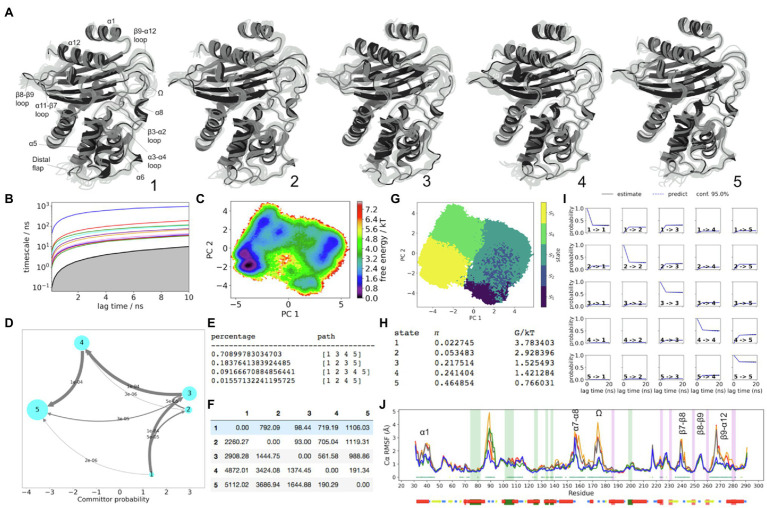
KPC-2 Dynamics. **(A)** The five metastable conformations sampled from the MSM. The ensemble of backbone geometries contained in each state is illustrated by displaying overlays of the most probable structure of the state (cartoon) on top the samples of the entire state (transparent lines) to highlight both the intrastate conformational variability and the interstate conformational differences. The highly dynamic loop regions have been labelled; **(B)** Implied timescale plot; **(C)** Free energy landscape; **(D)** Net Flux plot highlighting the probabilities of each transition in the relevant direction per unit time (5ns), between the highest energy state 1, and all other states. **(E)** Different pathways taken by trajectories when reaching state 5, having started in state 1, and the flux plot shows the probabilities of each transition in the relevant direction per unit time; **(F)** Mean first passage times between metastable states per ns; **(G)** Macrostate distributions of conformations projected onto the first two principal components (PC); **(H)** The population of each state (π) and its free energy estimates; **(I)** Chapman-Kolmogorov (CK) test plots and **(J)** Each RMSF line is based on Cα distances from the crystal structure conformation, as an average over 20 frames. The plot shows the positions of the α and β hydrophobic node residues within green and pink vertical bands, respectively. The small dots, where present, represent Kruskal Wallis ANOVA values of *p* <0.05.

From the MSM results, significant differences in structure attributed to each unique large FE minimum are observed by comparing the metastable trajectory frames sampled from the course-grained PCCA distributions. These conformations underlie the stationary distribution in a reversible Markov matrix, which has been lumped to explain dynamics between the most prominent FE minima. In turn, any significant differences between metastable conformations of hydrophobic node residues, as well as unique conformations of the loops, can be considered as evidence of significant structural coupling between dynamics of the loops and the regions of the hydrophobic core, as per locations on FE plot. The Cα-RMSF plots with Kruskal-Wallis ANOVA values of *p* <0.05 shown, indicate regions of the backbone where metastable dynamics is more pronounced (Figure 2).

### The Dynamics of KPC-2

The slowest dynamics in KPC-2 are recognizable from the Cα-RMSD plot where KPC-2 follows a large-scale double-well potential (Supplementary Figure S6). The regions in the structure, which are coupled upon transitioning between these two ensembles, are well resolved by the first PCA eigenvector (PC1), which separates states 1–3 apart from states 4 to 5 (Figure 2). As seen from the mean first passage times, this is the slowest dynamic process in KPC-2, which collectively describes cooperative conformational changes throughout the whole enzyme, albeit especially well in the α/β subdomain which are neighbouring the C69-C238 disulphide bond.

The backbone regions which are significantly involved in the double-well dynamics include the α1 helix (residues 32–47), β3-α2 loop (residues 66–73), α3-α4 loop (residues 102–108), α5-α6 loop (residues 129–132), α6 helix (including both α6 hydrophobic nodes), α7-α8 loop (residues 158–164), the distal region of α8 and the Ω-loop (residues 171–180), backbone region between the Ω-loop and α9 helix (residue 180–187), β7-β8 loop partially involving both strands (residues 235–247), and β9-α12 loop with the end of β9 strand (residues 262–279).

The dynamics of the distal flap (residues 86–93) are explained primarily by PC2, where states 1 and 5 show this loop visiting a particularly stable conformation, analogous to that which is seen in the crystal structure (Figure 2). The metastability of the distal flap was described to involve “open” and “closed” conformations. The open conformation of this highly mobile loop was linked with opening of a cryptic pocket at the distal end of the α-helical sub-domain, granting access to α2 and α5 hydrophobic nodes (Galdadas et al., 2018). To confirm that states 1 and 5 indeed describe the open-flap conformation, the distance between G89(Cα) and A201(Cα) was measured, which presented two peaks (Supplementary Figure S7A). The prevalence of the open conformation, where the distance between G89(Cα) and A201(Cα) is higher (~17.5Å) was approximately 1.6-fold higher than closed (~14Å). The G89(Cα) atom visits a particularly stable open conformation only in states 1 and 5, while in the other states (2–4), the distal flap is in a closed conformation (Supplementary Figure S7).

The distal flap is the most flexiible loop within the α-helical subdomain of KPC-2, as seen from the RMSD and RMSF results (Supplementary Figures S4 and S5). To visualise the relative involvement of the distal flap in the global dynamics of KPC-2, a 2D information map from a normalised mutual information (MI) distance matrix was built (Supplementary Figure S8), which indicated that the distal flap along with the Ω-loop and the α7-α8 loop (residues 155–159) collectively form a trio of loops that describe the most informative subset of dynamic correlations within the system. At the centre of this map are the structures which are in the direct neighbourhood of the disulphide bond and which significantly obey the longest-distance dynamics of the double-well (e.g., including the β7-β8 loop, β9-α12 loop, and the Ω-loop). Further out, the map shows structures that may share dynamic coupling at faster timescales. The α3-α4 loop appears on the edge of this map adjacent to the α6 node, which is nearest to the α5-α6 loop. The RMSF plots show metastability in the α5-α6 and the α3-α4 loops as being well described by the double-well potential (Figure 2J and Supplementary Figure S5). However, the side chain conformation of W105 (located at the tip of α3-α4 loop) is not well resolved in PCCA samples of states 1–3 vs. states 4–5. It is worth emphasising that the W105 side chain conformation was anticipated to be highly represented by the conformation of the α3-α4 loop and especially the W105(Cα) atom. LDA results (Supplementary Figure S9) illustrate this point in all four enzymes, highlighting correlations among the conformations of the α3-α4 loop, α5-α6 loops, and the α6 hydrophobic nodes. Moreover, the hydrophobic interactions between α5 and α6 nodes and α3-α4 loop node were anticipated to play a crucial role in the dynamics of W105, given that the mutations that can abolish these interactions, significantly alter the FE landscape of the W105 side chain in KPC-2 (Galdadas et al., 2018). Taken together, the lack of resolution of W105 by the MSM indicates that functionally significant dynamics in this region may occur at faster timescales and may not be resolved by the course grained 5-state MSM approach. Although, the slowest dynamic influence on α3-α4 loop may originate primarily from the α7-α8 loop, and arrive *via* the α6-α7 loop, α6 hydrophobic node, and the α5-α6 loop. It was also interesting to estimate how much meaningful correlation may exist between the W105 side chain and the distal flap, which is located >28Å away. The results show a weak positive correlation between only the highly stable “open” conformation (unique to states 1 and 5), and the “flipped-in” W105 conformation. Admittedly, this positive correlation is weak in magnitude, but may explain why α3-α4 loop is found near to the distal flap on the information map. In turn, it is likely that stabilization of the “open” conformation of the distal flap in states 1 and 5 can significantly modulate the allosteric landscape surrounding the α3-α4 loop at faster timescales, primarily by altering the hydrophobic interactions, which are present within the α-helical domain (between α2-α7, α5, and α6 helices). This observation is supported by pathways of signal propagation in KPC-2 that were recently reported by Galdadas et al. (2021).

The structure of the oxyanion hole is one of the hallmarks of β-lactamases (Pemberton et al., 2017; Cortina et al., 2018). Functionally, it is well recognised that the structure of the oxyanion hole, is described by the backbone of residue 237 and the amide of S70, and serves as a hydrogen bond donor to stabilize the negatively charged carbonyl oxygen of the β-lactam ring during acylation (upon formation of the first unstable acyl-enzyme tetrahedral covalent intermediate; Pemberton et al., 2017; Cortina et al., 2018). Moreover, the oxyanion hole may also need to be intact during ligand release. This occurs after S70 has recovered its proton and the side chain can rotate away from K73, towards the oxyanion hole (Pemberton et al., 2017; Supplementary Figure S1). It has been speculated that side chain dynamics of S70 may have a functional significance in carbapenemases (KPC-2 and SME-1), where S70 (along with other key residues) is structurally displaced closer to the entrance of the active site by 0.5–0.8Å compared to the active site topology in non-carbapenemases (Ke et al., 2007; Papp-Wallace et al., 2010; Fonseca et al., 2012; Naas et al., 2016). Dynamic considerations in this region of the active site are therefore important, given the fact that only carbapenemases maintain a highly conserved disulphide bond between C69 and C238, connecting the end of the β7 strand to the β3-α2 loop.

Functionally, the side chain at position at 237, due to its prominent location just above the active site on the β7 strand, may interact with carboxyl group in cephamycin and in carbapenem compounds, with likely significance for ligand binding and repositioning (Ke et al., 2007). This region appears highly conserved in both carbapenemases (KPC-2 and SME-1) and very different in SHV-1 and TEM-1, which both have hydrophobic A237 at this position instead.

The β7 strand backbone dynamics near residue 237 is additionally important due to its direct covalent relationship to the backbone near S70, *via* the C69-C238 disulphide bond. A constrained conformation of the β7 strand in KPC-2 is linked to the rotation of the ψ angle in C238, resulting in the flipping of the C238 backbone carbonyl group by ~180^°^ towards the active site in states 1, 2, and 3. Incidentally, in KPC-2, the slowest order parameter is the ψ angle of C238, where the rotameric state of this angle is directly coupled to the global double well in FE (Supplementary Figure S10). This angle is therefore strongly correlated with multiple structural changes throughout the enzyme and is related to significant differences in side chain conformations of residues in the active site, including T237, N170, H274, as well as S70 (at higher FE in state 1). Specifically, when the C238 backbone carboxyl group is rotated towards the active site in the higher FE states, the end of the β7 strand acquires a constrained (straightened) conformation directed towards the Ω-loop. This change causes T237 side chain to become displaced upwards (away from the centre of the active site), where it also appears less stable. On the other hand, the two of the lower FE states (states 4 and 5) represent relaxed β7 strand conformation where T237 side chain resembles the crystallographic conformation. Furthermore, in states 4 and 5, the aromatic side-chain of H274 (located on β9-α12 loop) also adopts a crystallographic pose, and can positively contribute to the stability of T237 side chain *via* van der Waals interaction with the methyl group. Moreover, the hydrogen bond between R220 side chain and the T237(Oγ) atom in states 4 and 5 is also collectively more stable, due to globally consistent high stability of the hinge region in KPC-2. Interestingly, within the dataset near state 1 there exists an even higher metastable FE state (at approximately 5.3G/kT), with relatively low occupancy at 300K, and is therefore not represented separately by this MSM model. In that state, the centre of mass of the entire disulphide bond experiences a metastable displacement, significantly effecting the conformation of the β3-α2 loop, which becomes “pushed in” towards the back of the active site. Such conformation was in turn linked with S70 side chain becomes highly unstable, as well as the oxyanion hole becoming consistently blocked by the backbone carbonyl oxygen of the C69, which also becomes rotated towards the active site.

In addition to the oxyanion hole to be intact, KPC-2 β-lactamase can only be catalytically efficient when the Ω-loop is stable. Moreover, the stable conformation of Ω-loop should not exceed 8Å distance between E166(Cδ) and N170(Cγ; Cortina et al., 2018). This criteria were calculated from the KPC-2 meropenem acyl-enzyme MD simulations (Cortina et al., 2018). Since our simulations were performed on the apo KPC-2 structure, we wanted to confirm that we sampled the conformations of the Ω-loop as described previously. Therefore, we measured the distance between E166(Cδ) and N170(Cγ). Collectively, 99.5% of frames were <8Å distance between these two atoms. In turn, the relative displacement of N170, E166, and S70 was not significantly sampled in the current trajectories. However, what appears to be clear from the current set of results is that the side chains (E166 and N170) are more stable when the entire Ω-loop is stable. The most catalytically permissive Ω-loop is observed in state 4, where the Ω-loop adopts a stable conformation, which is analogous to the crystal structure. This is also correlated to the apparent crystallographic conformation adopted by the α7-α8 loop (residues 155–159), which is a unique structural feature of state 4. Furthermore, state 4 also represented crystallographic conformations in the α4 helix, the adjoining α4-α5 region (residues 110–120), while the distal flap (in contrast) appeared the least stable in this state. Based on these MSM observations in addition to the presence of shared IC2 and/or IC3 RMSF peaks (Supplementary Figure S5), it appears that the four structural elements (Ω-loop, α7-α8 loop, the distal flap, and α4) are collectively coupled in a globally metastable manner.

The conformational drift plot in KPC-2 (Figure 2J) shows that seven hydrophobic nodes (located on the α3-α4 loop, and on helices α5, α6, α9, α11, and α12) represent statistically significant structural differences between the five sets of metastable MSM samples (values of *p*<0.05). In four of these nodes, which are all located in the α-network sub-domain between residues 100–140, the conformations appear to be mostly influenced by the global double-well dynamics, i.e., where states 1, 2, and 3 have different metastable conformations compared to states 4 and 5. The hydrophobic nodes located on helices α5 and α6 are important because the loop between them contains S130, which is involved during acylation, and N132, which is a hydrogen bond acceptor from K73. In states 1–3, the α5-α6 loop is gently shifted away from the centre of the active site (Figure 2). This shift is consistent with the global “double-well” dynamics described by PC1, and is caused by N-terminally directed metastable displacement of the entire α6 helix. The end of α6 helix, along with the adjoining α7 helix appear to involve more than one axis of variation, with the most noticeable shift in α7 helix observed in state 2. This unique conformation highlighted the appearance of a cryptic pocket between α2 and α7 helices in state 2 (Supplementary Figure S11; dark green pocket). This pocket is also the binding site of a phosphonic acid ligand in the crystal structure of KPC-2 (PDB id 6D18). Collectively, from these observations, it is therefore likely that α6-α7 loop is coupled primarily to the α7-α8 loop (residues 155–159), as well weakly to the distal flap. The latter connection may be largely significant at faster timescales (Galdadas et al., 2021), and is mediated by the hydrophobic contact between the α2 hydrophobic node residues (F75, L76, A79, and V80) and the α7 hydrophobic residues (L142, L148, F151, and M152).

Other pockets with volume>500Å^3^ are also identified (Supplementary Figure S11; orange and light green). The orange pocket appears adjacent to the Ω-loop, only in the low FE states 4 and 5, which is consistent with this loop dynamics in these states. The light green pocket has been reported previously by Galdadas et al. (2018) and is identifiable in the crystal structure (PDB id: 2OV5), and on average presented a relatively consistent volume in all metastable states. Occupancy and volume of this pocket may be slightly greater in transition states 2 and 3.

### The Dynamics of SME-1

**Figure 3:**
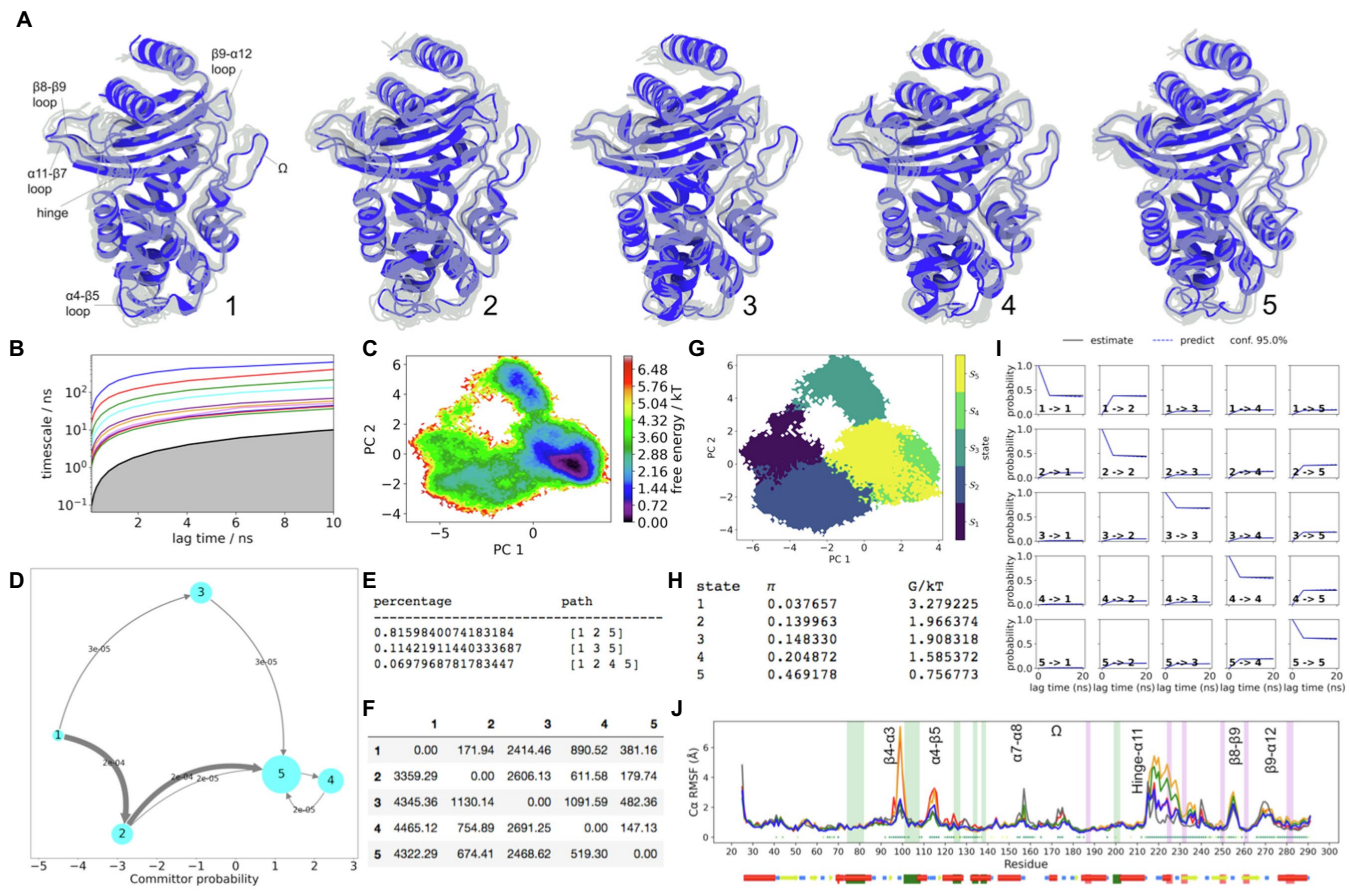
SME-1 Dynamics. **(A)** The five metastable conformations sampled from the MSM. The ensemble of backbone geometries contained in each state is illustrated by displaying overlays of the most probable structure of the state (cartoon) on top the samples of the entire state (transparent lines) to highlight both the intrastate conformational variability and the interstate conformational differences. The highly dynamic loop regions have been labelled; **(B)** Implied timescale plot; **(C)** Free energy landscape; and **(D)** Net Flux plot highlighting the probabilities of each transition in the relevant direction per unit time (5ns), between the highest energy state 1, and all other states. **(E)** Different pathways taken by trajectories when reaching state 5, having started in state 1, and the flux plot shows the probabilities of each transition in the relevant direction per unit time; **(F)** Mean first passage times between metastable states per ns; **(G)** Macrostate distributions of conformations projected onto the first two PC; **(H)** The population of each state (π) and its free energy estimates; **(I)** Chapman-Kolmogorov (CK) test plots and **(J)** Each RMSF line is based on Cα distances from the crystal structure conformation, as an average over 20 frames. The plot shows the positions of the α and β hydrophobic node residues within green and pink vertical bands, respectively. The small dots, where present, represent Kruskal Wallis ANOVA values of *p* <0.05.

Unlike in KPC-2, the distal flap in SME-1 presents no significant metastable dynamics (Figure 3). The dynamics of the α-helical subdomain is centred on the start of α3 and the end of α4 helices. The slowest dynamics within these regions clearly appears upon transition out of the higher energy states 1 and 2, towards the lower energy states. The transition states 3 and 4 being energetically similar are structurally very different in terms of conformation of the α11 helix and the hinge region (Figure 3).

The longest mean first passage time during the net flux pathways is from states 1 to 3 (Figure 3). This means that the FE barrier between these states is high. This is also seen from the reweighted FE surface. The flux pathway that visits state 3 has only ~11% prevalence at equilibrium. On the other hand, the decrease in FE starting from the source (state 1) to the sink (state 5), predominantly visits state 2 (~89% of the time). Collectively, this indicates that prominent conformational changes of the hinge region may have a functional significance in SME-1. When the hinge region is retracted away from the active site, as in states 2 and 4, the hydrogen bond from R220 to S237(Oγ) is lost. This allows the S237 side chain to rotate towards H274, forming a new hydrogen bond, which stabilizes S237. Similar to KPC-2, this highly stable orientation of the S237 side-chain appears to be strongly coupled to the C238 backbone carbonyl group, which is rotated away from the active site in a metastable manner (in states 2, 4, and 5). Inspite of this similarity, the end of the β7 strand may span significantly shorter kinetic distances than in KPC-2. This is seen from the filtered Cα-RMSF plots (Supplementary Figure S5A), where both regions involved with the C69-C238 disulphide bond are better described by multiple faster eigenvectors compared to KPC-2. The blue line (SME-1) of the standard RMSF plot (Supplementary Figure S5B) shows shorter deviations away from crystal structure conformation, compared to KPC-2 (black line). The role of the disulphide bone in the FE landscape of SME-1 is significant, as indicated by mutual information peaks (Supplementary Figure S8B) in both residues (C69 an C238). This is supported by the experimental observation that the stability of the disulphide bond has been known to be essential for catalytic function in SME-1 (Majiduddin and Palzkill, 2003).

In state 5, the conformation of the Ω-loop remains close to the stable crystallographic conformation where the N170 side chain is not perturbed. The α7-α8 loop (residue 156–159) displays similar dynamics in SME-1, as in KPC-2, where the crystallographic conformation is linked to greater stability in the Ω-loop. In SME-1, only state 3 presents the metastable downwards position in this loop (i.e., opposite to the crystallographic pose), which is linked to unfavourable instability of the Ω-loop.

In contrast to KPC-2, the hydrophobic α-network in SME-1 is less metastable, as seen from the lack of statistically significant differences between the Cα-RMSF values in regions highlighted by green boxes (Figure 3J). The absence of slow dynamics in these regions is further supported by the results of the filtered RMSF (Supplementary Figure S5A). However, there is significant state separation captured by the PCCA samples involving the α5-α6 loop, which is displaced away from the centre of the active site in states 1 and 3. This deviation is associated with a metastable shift in the α5 helix, and is only sampled in states 1 and 3. This is likely the cause for increased instability of S130 side chain in these two states (Figure 3).

Within the α/β-subdomain, the two hydrophobic nodes surrounding the α11-β7 loop (residues 224–233) are significantly coupled to the slow dynamics in this region. In state 2, as a result of significant change in K234 backbone (β7 strand) conformation, the hydrogen bond between K234 and S130 side chains is broken. Moreover, as noted previously, the conformation of the α5-α6 loop in state 2 is comparable to that observed in the crystal structure. Collectively, these two spatially separate backbone features allow S130 side chain to experience a relatively stable conformation towards the active site where it can be available during acylation. It can be seen from the filtered RMSF plot (Supplementary Figure S5A), that IC3 simultaneously describes both of these backbone regions. Moreover, IC3 most significantly describes the α11 helix and is also largely involved in metastability of the α3 helix. In state 2, H105 side chain is most readily found at the “flipped-in” position, which can promote ligand access. Cooperative dynamic changes in the backbone of S130, as well as changes in side chain conformation at position 105 and K73, have been previously observed experimentally when the active site is occupied by a ligand (cefotaxime) in Toho-1, involving S130(γO) movement closer to K73, while K73(Nζ) can move closer to S70 (Shimamura et al., 2002; Langan et al., 2018). Similar movements in W105 and S130 have been observed in apo vs. holo crystalline forms of KPC-2 (Ke et al., 2007; Papp-Wallace et al., 2010).

The metastable coupling of the polar H105 side chain to the overall FE landscape in SME-1 is not well resolved by the MSM. LDA results show that the dynamics of this side chain is significantly different from the other three enzymes (Supplementary Figures S9A,D,E). For example, there is relatively minimal bias between “flipped-out” vs. “flipped-in” conformation visited by the side chain at equilibrium, as shown by 51.2 and 48.8% prevalence values (Supplementary Figure S9A). In fact, the 3D distribution of H105(Cα) atom cannot be distinguished as metastable, and likewise the H105(Cγ) atom when taken alone also follows a continuous unimodal (ellipsoidal) distribution. This comes in complete contrast to the three other enzymes when sampled at equilibrium; perhaps due to chemistry of this side chain being polar.

Pockets were also identified in SME-1, at positions similar to that in KPC-2 (Supplementary Figure S11). The appearance of the pocket located between α2 and α7 helices (dark green) was significantly higher in all five SME-1 metastable states compared to KPC-2, yet on average, the volume of these pockets is smaller than that observed in KPC-2. The persistence rate for the pocket located adjacent to the Ω-loop (orange) was also consistently higher in SME-1 compared to KPC-2. On average, this pocket remains >600Å^3^ in volume and is observed in all five metastable states.

### The Dynamics of Non-Carbapenemases: TEM-1 and SHV-1

The stability of the hinge region in non-carbapenemases (TEM-1 and SHV-1) has known importance for stabilising substrate’s C3-carboxylate-group *via* hydrogen bond from a conserved non-catalytic water molecule, which is often found anchored between backbone carbonyl of V216 and the side chain guanidinium group of R244 (Wang et al., 2002). For example, SHV-1-meropenem (pdb ID: 2ZD8) and TEM-1-iminpenem (pdb ID: 1BT5) acyl-enzymes show this interaction, where the non-catalytic water molecule contributed towards desirable, but in the case of these ligands, catalytically unfavourable pose away from the catalytic water molecule.

**Figure 4:**
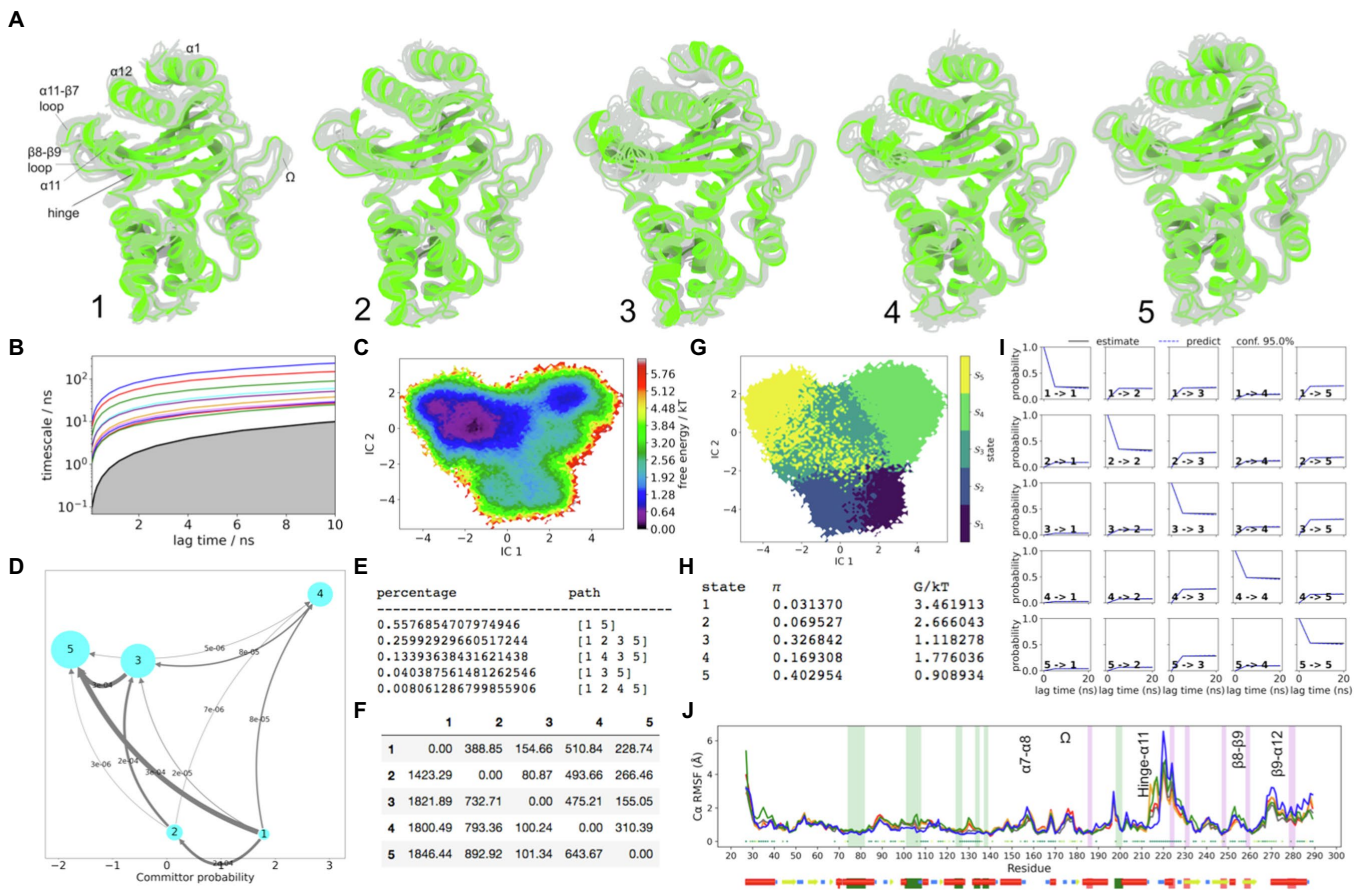
TEM-1 Dynamics. **(A)** The five metastable conformations sampled from the MSM. The ensemble of backbone geometries contained in each state is illustrated by displaying overlays of the most probable structure of the state (cartoon) on top the samples of the entire state (transparent lines) to highlight both the intrastate conformational variability and the interstate conformational differences. The highly dynamic loop regions have been labelled; **(B)** Implied timescale plot; **(C)** Free energy landscape; and **(D)** Net Flux plot highlighting the probabilities of each transition in the relevant direction per unit time (5ns), between the highest energy state 1, and all other states. **(E)** Different pathways taken by trajectories when reaching state 5, having started in state 1, and the flux plot shows the probabilities of each transition in the relevant direction per unit time; **(F)** Mean first passage times between metastable states per ns; **(G)** Macrostate distributions of conformations projected onto the first two PC; **(H)** The population of each state (π) and its free energy estimates; **(I)** Chapman-Kolmogorov (CK) test plots and **(J)** Each RMSF line is based on Cα distances from the crystal structure conformation, as an average over 20 frames. The plot shows the positions of the α and β hydrophobic node residues within green and pink vertical bands, respectively. The small dots, where present, represent Kruskal Wallis ANOVA values of *p*<0.05.

R244 is located at the start of the β8 strand. Unlike V216 (hinge region), this residue displays a stable conformation in all of the sampled states in TEM-1. Based on the current model of TEM-1, the α11 helix does not converge back towards the topology conformation even in the lowest FE state 5. Nevertheless, the hinge region was resolved relatively well. In state 5, the hinge region is stable, which is accompanied by catalytically favourable conformations of various other residues of the active site. For example, the S130 side chain is stable because the backbone of the α5-α6 loop experiences the shortest displacement away from the crystallographic conformation (Figure 4). The K73 side chain stabilizes near the hydroxyl group of S70, and Y105 presents more conformations where the flipped-out conformation (catalytically active) is seen. In state 4, the side chain of Y105 visits the bulk. This is associated with increased instability of the α3-α4 loop and is coupled to the displacement of the α4 helix along with the displacement observed in the α5-α6 loop and the α5, α6 hydrophobic nodes, which surround it. The hinge region in state 4 is directed way from the active site and is unstable. The K73 side chain is displaced laterally relative to the centre of the enzyme, with accompanied instability. The S130 side chain moves away from the active site and is also highly unstable. Clearly state 4 cannot be catalytically efficient, although less than 14% of flux may visit state 4 at equilibrium (Figure 4).

In TEM-1, the α-helical subdomain is relatively stable, with no kinetic changes observed in the C77-C123 bond, which connects helices α2-α5. However within α5 and α6 helices, as in the case of carbapenemase enzymes (KPC-2 and SME-1), there are significant and conserved metastable traits, involving the two hydrophobic nodes that surround the α5-α6 loop. As in SME-1, TEM-1 displays significant metastability among the nodes of the α/β-subdomain including α11 and β8 nodes. The residues in α11 and β8 nodes are in direct hydrophobic contact with each other. Furthermore, like in KPC-2 and SME-1, the metastability of the hydrophobic nodes in α5 and α6 helices is directly coupled to the instability of the S130 side chain and is also indirectly coupled to other significant perturbations at the active site (e.g., the destabilization of the hydrophobic interactions with α3-α4 loop).

The most prominent allosteric pocket identified on TEM-1 is between α11 and α12 helices (Supplementary Figure S11; violet pocket). The α11-α12 region is a well-documented allosteric site in TEM-1 (Horn and Shoichet, 2004). Ligand binding in this site abolishes the necessary packing interactions from L220 (α11 helix) to N276 (α12 helix), which otherwise function to stabilize R244. This was also dynamically observed in simulations, which confirmed that ligand binding at the cryptic pockets between α11 and α12 helices caused significant changes in rotameric states of N276 and R244 side chains. Furthermore, the allosteric site formed between α11 and α12 helices is at least partially open 53% of the simulation time. However, this prevalence can be significantly increased by the presence of a small molecule (Bowman and Geissler, 2012). Moreover all of the major pockets described collectively (Bowman and Geissler, 2012; Bowman et al., 2015) were also sampled in the current TEM-1 simulation.

The flexibility of the Ω-loop in TEM-1 at μs timescales may be necessary for substrate gating, but at ps timescales, the basal stability of this loop is certainly advantageous to reduce solvent exposure of the bound ligand (Fisette et al., 2012; Meneksedag et al., 2013; Hart et al., 2016). For example, the slow dynamics was identified in inhibitor resistant mutation of TEM-1 (M69L), where a change in long timescale motions of the Ω-loop was associated with significant change in electrostatic and van der Waals components of free energy. This resulted in elevated binding FE for inhibitors (clavulanate, sulfabactam, and tazobactam), causing significant decrease in binding affinity for these compounds, but not for five different β-lactam substrates tested (Meroueh et al., 2002). Furthermore, an increased stability of the Ω-loop as well as the α3-α4 loop in TEM-1 (*via* naturally occurring E104K and G238S mutations) has been experimentally linked with 1,400-fold increase in cefotaxime hydrolysis efficiency, and 500-fold increase in minimum inhibitory concentration of *E. Coli*, i.e., ESBL phenotype in TEM (Hart et al., 2016). G238S mutation is located on the C-terminus of the β7 strand and stabilizes the Ω-loop by hydrogen bonds towards N170 side chain and backbone, while E104K can interact with P167 from the other side of the loop. It is likely that any mutation, which stabilizes the proximal end of the Ω-loop from the α3-α4 direction, can favourably stabilize both loops at the same time. Unlike in MSMs of wild type and mutant (E104K/G238S) TEM-1 presented by Hart et al. (2016), in the current dataset, there was no major deviation away from the reference crystal structure, which would significantly involve the entire α8 helix. Only at the highest energy state 1, the N-terminus of α8 helix can be seen directed towards the active site.

**Figure 5:**
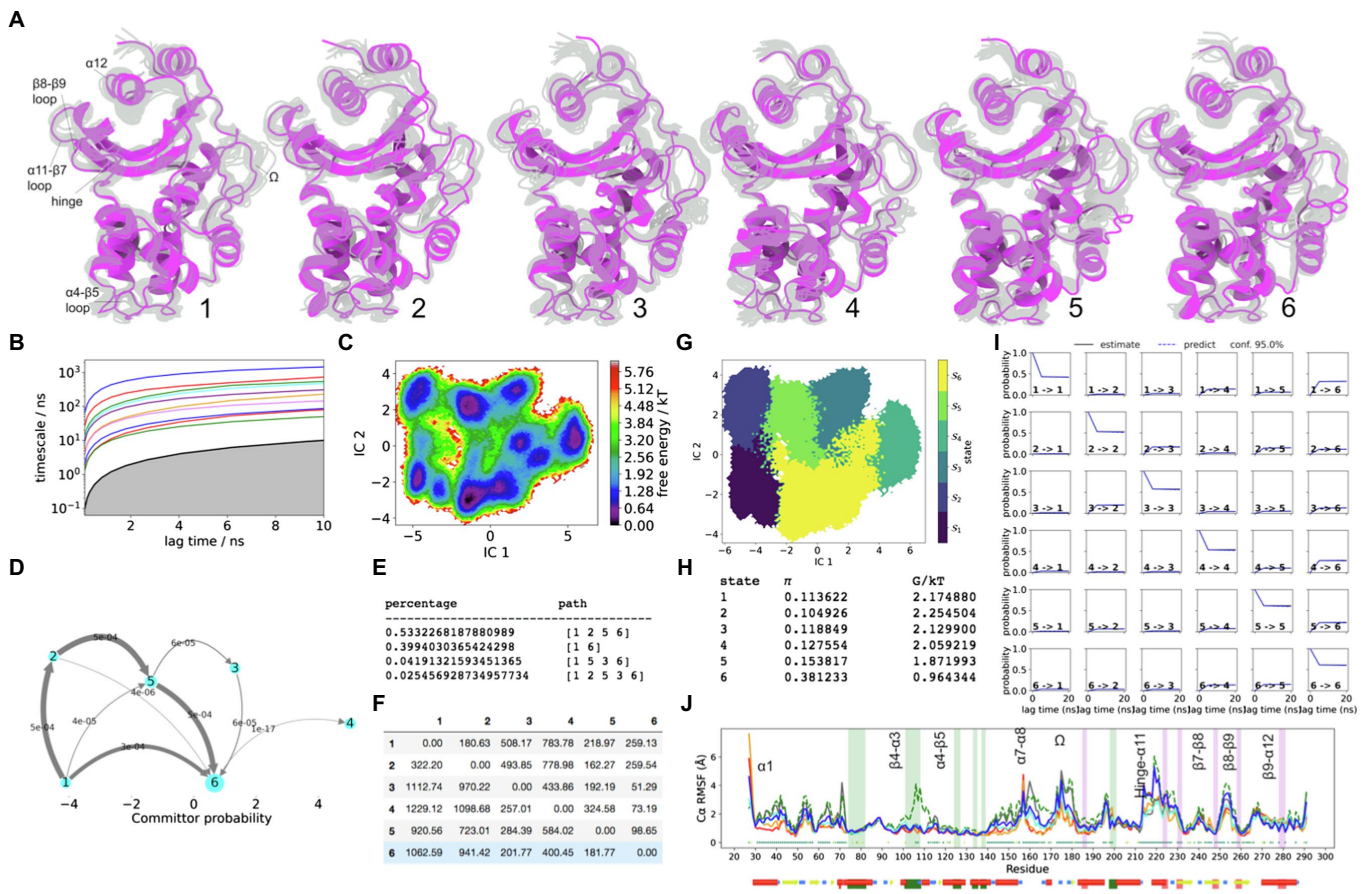
SHV-1 Dynamics. **(A)** The five metastable conformations sampled from the MSM. The ensemble of backbone geometries contained in each state is illustrated by displaying overlays of the most probable structure of the state (cartoon) on top the samples of the entire state (transparent lines) to highlight both the intrastate conformational variability and the interstate conformational differences. The highly dynamic loop regions have been labelled; **(B)** Implied timescale plot; **(C)** Free energy landscape; and **(D)** Net Flux plot highlighting the probabilities of each transition in the relevant direction per unit time (5ns), between the highest energy state 1, and all other states. **(E)** Different pathways taken by trajectories when reaching state 5, having started in state 1, and the flux plot shows the probabilities of each transition in the relevant direction per unit time; **(F)** Mean first passage times between metastable states per ns; **(G)** Macrostate distributions of conformations projected onto the first two PC; **(H)** The population of each state (π) and its free energy estimates; **(I)** Chapman-Kolmogorov (CK) test plots and **(J)** Each RMSF line is based on Cα distances from the crystal structure conformation, as an average over 20 frames. The plot shows the positions of the α and β hydrophobic node residues within green and pink vertical bands, respectively. The small dots, where present, represent Kruskal Wallis ANOVA values of *p* <0.05.

The FE landscape of SHV-1 is very wide and relatively non-specific. For example, the first PCA eigenvalue in SHV-1 is almost double than of the other three enzymes. The fraction % of Cα atoms with RMSD >1Å in SHV-1 is more than triple that of TEM-1 (Supplementary Figure S4). All FE minima are relatively uniform in prevalence, compared to the other enzymes, yet the mean first passage times along the major net flux pathways largely reside in triple digits, in contrast to the other enzymes (Figure 5).

Analogous to TEM-1, there exists one metastable state in SHV-1, which is characterised by α3-α4 displacement away from the core, among other unique conformations at the active site (e.g., S70 being displaced to the top of the active site as a result of β3-α2 loop rotation). This is observed in state 4 (Figure 5). Since no significant net flux is directed towards state 4, it remains relatively redundant at equilibrium conditions, and therefore it was omitted from the Kruskal Wallis ANOVA significance test (Figure 5J). State 3 presents analogous metastability of S70 backbone, but less than 8% of flux may visit state 3. Approximately, 92% of net flux between the highest FE (state 1) and the lower FE sink (state 6) do not visit states 3 or 4 because the local FE barriers to enter those minima are steep (Figure 5). Interestingly, among these large scale kinetic transitions in SHV-1, the expected α-network hydrophobic nodes do not display significant metastability based on the conformational drift RMSF plot (Figure 5).

Both non-carbapenemases (SHV-1 and TME-1) presented comparable consistency in locations of cryptic sites (Supplementary Figure S12). Due to the greater flexibility of the secondary structure in SHV-1, the prevalence of these pockets was greater than in TEM-1. In both enzymes, the light blue and cyan pockets are located in solvent inaccessible core. The most significant observation of this study involves the presence of dark green and orange pockets, which were consistently identified in all four enzymes.

### Convolutional Variational Autoencoder Based Deep Learning

The residues of the hydrophobic network in Class A β-lactamases comprise of a highly stable core, where dense van der Waals (packing) interactions physically prevent long distance movements. Backbone dynamics in many of these residues, resides under Gaussian stationary distribution, when looking at either the cartesian coordinates of Cα atom, or the backbone dihedral angles of specific residues. This indicates that the thermal vibrations at fast timescales may significantly obscure the underlying (potentially important) conformational signals. Moreover, multivariate distributions with Gaussian marginals are not necessarily Gaussian. Therefore, just because all of the raw input features cannot be collectively resolved by standard unsupervised linear methods did not imply that any of the features are redundant. In fact, the necessity to keep all of hydrophobic node residues for the analysis of their dynamics is highlighted by the immediate off-diagonal elements in the mutual information matrices, indicating strong correlation in backbone dynamics especially between immediately neighbouring residues (relaying signals). This is illustrated in Supplementary Figure S8, showing non-zero average mutual information present within the hydrophobic nodes, even when course-grained (π/5 radian) discretisation was used. Certainly many of the nodes do not initiate dynamics, however, they are involved in propagating allosteric signals, which are initiated between the loops.

The backbone conformations sampled by the Cα atoms of the hydrophobic nodes were featurised *via* 48 by 48 symmetric distance matrices, and the dimensionality reduction of the hydrophobic node dynamics was approached using unsupervised 2D image clustering algorithm (CVAE). The use of 64 filters in each of the four convolutional layers, and no pooling layers maintained a deep representational space of trainable parameters in each hidden layer.

**Figure 6:**
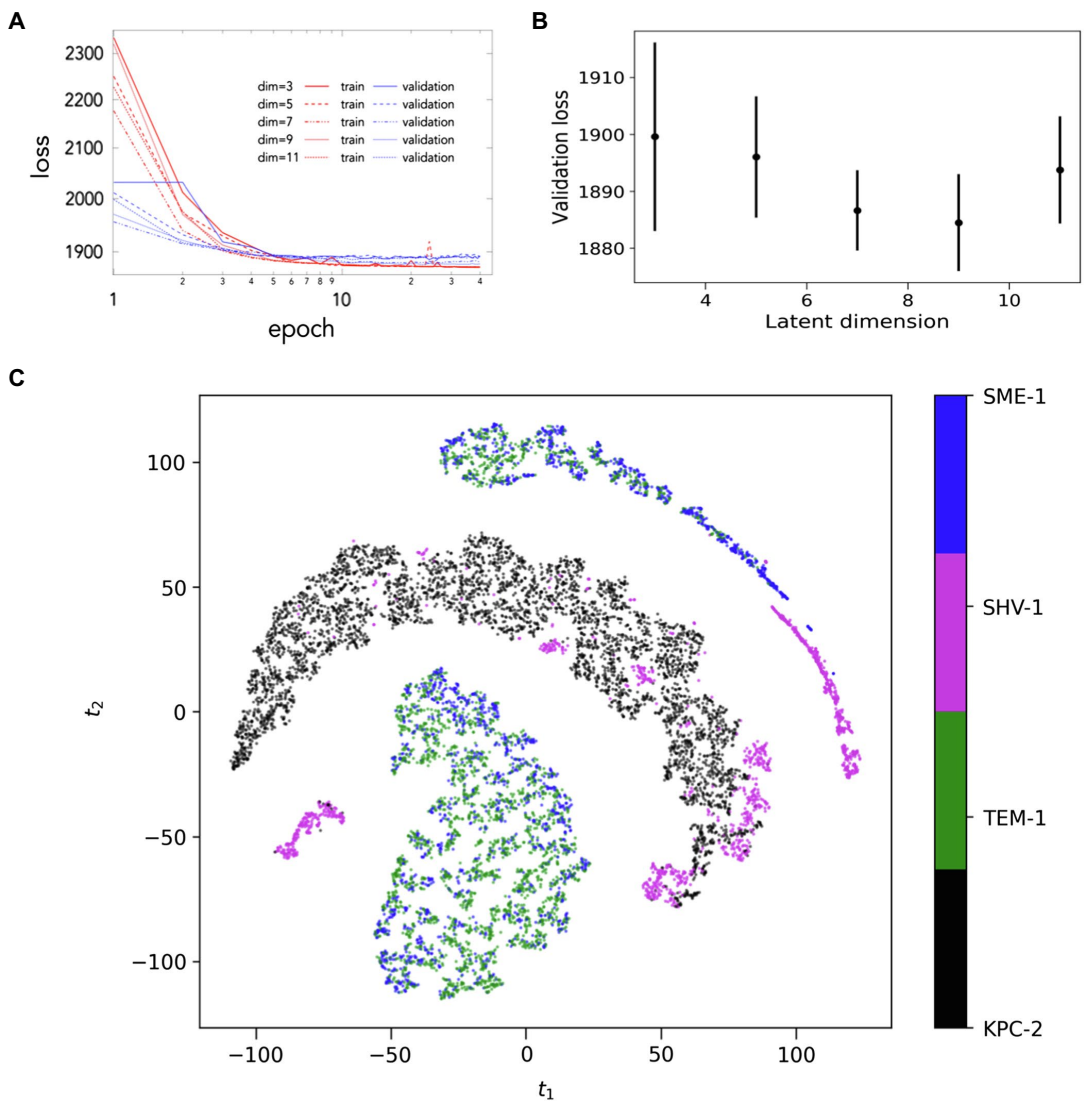
Convolutational Variational Autoencoder (CVAE)-based Deep learning analysis. **(A)** The validation loss during CVAE implementation is plotted at different latent dimension for determining optimum values of the low dimension; **(B)** the training and validation loss is plotted as\ assessed simultaneously over consecutive epochs at various reduced dimensions; and **(C)** the low dimensional latent space of CVAE model’s learnt features of the original high dimensional input data as represented in two dimensions. The original high dimensional data are transformed into distance matrix format during pre-processing that is then fed into the CVAE architecture. The CVAE then captures the intrinsic features of the original high dimensional data in order to best describe the original system. This CVAE captured information is processed in two-dimensional representation following t-sne implementation. The results show that the dynamics of SME-1 and TEM-1 is distinct from KPC-2 and features of SHV-1 dynamics are comparable to all other enzymes.

As a first step to determine CVAE learning quality of the given simulations, the trajectories from all systems were mixed together followed by the evaluation of the training and validation loss of the combined dataset. The CVAE model was implemented and tested from reduced dimension of 3–11 (Figure 6A). Both, the training and the validation loss decrease over the number of epochs trained as expected while validation loss is slightly higher than training loss. This conforms the normal behaviour. During this step, with decreasing dimension size of the reduced latent space, the corresponding input data are compressed by utilising the model’s more representation capability. Gradually, once this reduced latent dimension size becomes too small compared to the model’s architecture, it would start over-fit local features while introducing additional noise. Here the regularising term (Kullback–Leibler or KL divergence) of the loss function becomes much important. Gradually, the overall loss value attains an optimal value. This optimum value is in between those two extremes. For the dataset considered here, the implemented CVAE model is remarkably stable and robust. This is confirmed from the fact that the validation loss remains close during the various latent dimensions (Figure 6B). Based on this, latent dimension 7 was selected for the lowest dimension where the loss value is small and simultaneously the uncertainty in loss is also lower while being compared to other dimensions. This is also confirmed from the loss behaviour with epochs.

Next, *t*-distributed stochastic neighbour embedding (t-sne) on the compressed reduced-dimension data was implemented. This is performed in order to better visualize the compressed data in simplified two dimensions. Figure 6C shows the two-dimensional t-sne representation of the compressed CVAE low-dimension data. The combined CVAE-tsne representation is able to cluster the four different systems based on their local and global conformational dynamics as they are evolved with the simulation trajectories.

The t-sne visualisation of the CVAE latent space plots the conformations sampled by the hydrophobic network in the four systems (Figure 6C). These conformations are resolved based on the dynamics generated by distance matrix of the hydrophobic nodes. SME-1 and TEM-1 share similar dynamics, dominated by the motions in the hinge region. The conformations of the KPC-2 enzyme are resolved separately, driven by the motions in the distal flap, α7-α8 loop, Ω-loop, β7-β8 loop, and β9-α12 loops. In SHV-1, where motions similar to those observed in KPC-2, SME-1 and TEM-1 are all observed, the conformations are clustered based on the similar dynamics. The deep learning results are consistent with the observations from MSMs, auto-correlated flexibility profiles, and mutual information. It is worth emphasising that the dynamics featurised on the distance matrices of the hydrophobic nodes alone cannot resolve the systems into carbapenemases or non-carbapenemases, suggesting that other features might also contribute to the complex dynamics of these enzymes.

The importance of residue at position 105 has been described previously by Bonomo and co-workers (Papp-Wallace et al., 2010, 105). However, no link between the hydrophobic nodes and residue 105 has been reported. The presence or absence of correlation between the hydrophobic network and residue 105 was also assessed in each system (Supplementary Figures S13 and S14). CV1 represents W105 in “flipped-in” conformation (side chain pointing towards L167). This was resolved only in KPC-2, TEM-1, and SHV-1, but not in SME-1. This unsupervised result agrees with earlier discussion about H105 in SME-1, where the side chain is polar. CV2 represents W105 in “flipped-out” conformation (side chain pointing towards T216). This was resolved in all four systems, but should be considered alongside the fact that T216 (hinge region) is highly mobile in SME-1 and TEM-1. In SME-1, the shape of the CV2 and CV3 distributions are similar, and the embedding colourings for CV2 and CV3 also appear similar. It can be concluded that the hydrophobic network in SME-1 is strongly coupled to the dynamics of the hinge region. The same can be said about TEM-1, but not KPC-2. The hinge region was stable in KPC2. In TEM-1 and SHV-1, an additional 3rd region of the embedding is coloured with higher distances in CV1 and CV2. This is Y105 conformation where the side chain is directed towards the bulk in these enzymes.

## Conclusion

In this study, we report on the role of hydrophobic nodes in the dynamics of four class A β-lactamase enzymes including KPC-2, SME-1, SHV-1, and TEM-1. It is clear from the analysis that the hydrophobic interactions between α5 and α6 nodes and the hydrophobic residues of α3-α4 loop are an important mechanism by which metastable signal is relayed from the global FE landscape towards the functionally significant side chain at position 105. This has been experimentally demonstrated by us previously (Galdadas et al., 2018) and is in accordance with the cross coupling interactions between the variables of interest and the catalytically important regions like the Ω loop, the hinge region, distal flap, and the α3-α4 loop. Our results collectively suggest that the class A enzymes described here, share dynamic similarities. This explains why some mutations, far from the active site that can alter dynamics have the ability to change substrate profiles of these enzyme.

## Data Availability Statement

The raw data supporting the conclusions of this article will be made available by the authors, without undue reservation.

## Author Contributions

This work has been adapted from the MSc dissertation of EO. EO, AP, and SH: simulations. EO, JY, DB, and SH: data analysis. EO, GF, RB, DB, and SH: manuscript writing. All co-authors: other inputs. All authors contributed to the article and approved the submitted version.

## Funding

SH and RB acknowledge a grant from the National Institutes of Health United States under the award number RO1AI063517. This material is based upon work supported by the U.S. Department of Energy, Office of Science, Office of Advanced Scientific Computing Research, under contract number DEAC05- 00OR22725. This research is sponsored in part by the Laboratory Directed Research and Development Program of Oak Ridge National Laboratory, managed by UT-Battelle, LLC, for the U.S. Department of Energy. This research used resources of the Oak Ridge Leadership Computing Facility at the Oak Ridge National Laboratory, which is supported by the Office of Science of the U.S. Department of Energy under Contract no. DE-AC05-00OR22725.

## Licenses and Permissions

This manuscript has been co-authored by UT-Battelle, LLC under Contract No. DE-AC05-00OR22725 with the United States Department of Energy. The United States Government retains and the publisher, by accepting the article for publication, acknowledges that the United States Government retains a non-exclusive, paidup, irrevocable, world-wide license to publish, or reproduce the published form of this manuscript, or allow others to do so, for United States Government purposes. The Department of Energy will provide public access to these results of federally sponsored research in accordance with the DOE Public Access Plan (http://energy.gov/downloads/doe-public-access-plan).

## Conflict of Interest

The authors declare that the research was conducted in the absence of any commercial or financial relationships that could be construed as a potential conflict of interest.

## Publisher’s Note

All claims expressed in this article are solely those of the authors and do not necessarily represent those of their affiliated organizations, or those of the publisher, the editors and the reviewers. Any product that may be evaluated in this article, or claim that may be made by its manufacturer, is not guaranteed or endorsed by the publisher.

## Footnotes

^1^ https://github.com/bieniekmateusz/pymol-mdanalysis

## References

[ref1] Abraham M. J. Murtola T. Schulz R. Páll S. Smith J. C. Hess B.. (2015). GROMACS: high performance molecular simulations through multi-level parallelism from laptops to supercomputers. SoftwareX 1, 19–25. doi: 10.1016/j.softx.2015.06.001

[ref2] Akere A. Chen S. H. Liu X. Chen Y. Dantu S. C. Pandini A.. (2020). Structure-based enzyme engineering improves donor-substrate recognition of Arabidopsis thaliana glycosyltransferases. Biochem. J. 477, 2791–2805. doi: 10.1042/BCJ20200477, PMID: 32657326 PMC7419078

[ref3] Arnold R. S. Thom K. A. Sharma S. Phillips M. Kristie Johnson J. Morgan D. J. (2011). Emergence of *Klebsiella pneumoniae* Carbapenemase-producing bacteria. South. Med. J. 104, 40–45. doi: 10.1097/SMJ.0b013e3181fd7d5a 21119555 PMC3075864

[ref4] Avcı F. Altınışık Kaya F. E. Vardar Ulu D. Ozkirimli E. Sariyar Akbulut B. (2016). An evolutionarily conserved allosteric site modulates beta-lactamase activity. J. Enzyme Inhib. Med. Chem. 31, 33–40. doi: 10.1080/14756366.2016.1201813, PMID: 27353461

[ref5] Bhowmik D. Gao S. Young M. T. Ramanathan A. (2018). Deep clustering of protein folding simulations. BMC Bioinformatics 19:484. doi: 10.1186/s12859-018-2507-5 30577777 PMC6302667

[ref6] Bowman G. R. Bolin E. R. Hart K. M. Maguire B. C. Marqusee S. (2015). Discovery of multiple hidden allosteric sites by combining Markov state models and experiments. Proc. Natl. Acad. Sci. U. S. A. 112, 2734–2739. doi: 10.1073/pnas.1417811112, PMID: 25730859 PMC4352775

[ref7] Bowman G. R. Geissler P. L. (2012). Equilibrium fluctuations of a single folded protein reveal a multitude of potential cryptic allosteric sites. Proc. Natl. Acad. Sci. 109, 11681–11686. doi: 10.1073/pnas.1209309109, PMID: 22753506 PMC3406870

[ref8] Bush K. (2018). Past and present perspectives on β-lactamases. Antimicrob. Agents Chemother. 62:e01076-18. doi: 10.1128/AAC.01076-18, PMID: 30061284 PMC6153792

[ref9] Bush K. Bradford P. A. (2016). β-Lactams and β-lactamase inhibitors: an overview. Cold Spring Harb. Perspect. Med. 6:a025247. doi: 10.1101/cshperspect.a025247 27329032 PMC4968164

[ref10] Cho E. Rosa M. Anjum R. Mehmood S. Soban M. Mujtaba M.. (2021). Structural dynamics of the β-coronavirus 3CL Mpro protease ligand binding sites. Biophysics 61, 3058–3073. doi: 10.1101/2021.03.31.437918 34124899

[ref11] Cho J. C. Zmarlicka M. T. Shaeer K. M. Pardo J. (2018). Meropenem/Vaborbactam, the first Carbapenem/β-lactamase inhibitor combination. Ann. Pharmacother. 52, 769–779. doi: 10.1177/1060028018763288, PMID: 29514462

[ref12] Chudyk E. I. Limb M. A. L. Jones C. Spencer J. van der Kamp M. W. Mulholland A. J. (2014). QM/MM simulations as an assay for carbapenemase activity in class A β-lactamases. Chem. Commun. 50, 14736–14739. doi: 10.1039/C4CC06495J 25321894

[ref13] Cortina G. A. Hays J. M. Kasson P. M. (2018). Conformational intermediate That controls KPC-2 catalysis and Beta-lactam drug resistance. ACS Catal. 8, 2741–2747. doi: 10.1021/acscatal.7b03832, PMID: 30637173 PMC6324736

[ref14] Daniel W. W. (1990). Applied Nonparametric Statistics. 2nd *Edn*. Boston: PWS-KENT.

[ref15] Davies J. Davies D. (2010). Origins and evolution of antibiotic resistance. Microbiol. Mol. Biol. Rev. 74, 417–433. doi: 10.1128/MMBR.00016-10, PMID: 20805405 PMC2937522

[ref16] Doerr S. Harvey M. J. Noé F. De Fabritiis G. (2016). HTMD: high-throughput molecular dynamics for molecular discovery. J. Chem. Theory Comput. 12, 1845–1852. doi: 10.1021/acs.jctc.6b00049, PMID: 26949976

[ref17] Dolk F. C. K. Pouwels K. B. Smith D. R. M. Robotham J. V. Smieszek T. (2018). Antibiotics in primary care in England: which antibiotics are prescribed and for which conditions? J. Antimicrob. Chemother. 73, ii2–ii10. doi: 10.1093/jac/dkx504 29490062 PMC5890730

[ref18] Drawz S. M. Bonomo R. A. (2010). Three decades of β-lactamase inhibitors. CMR 23, 160–201. doi: 10.1128/CMR.00037-09 PMC2806661 20065329

[ref19] Feenstra K. A. Hess B. Berendsen H. (1999). Improving efficiency of large time-scale molecular dynamics simulations of hydrogen-rich systems. J. Comput. Chem. 20, 786–798. doi: 10.1002/(SICI)1096-987X(199906)20:8<786::AID-JCC5>3.0.CO;2-B 35619462

[ref20] Fisette O. Gagné S. Lagüe P. (2012). Molecular dynamics of class A β-lactamases—effects of substrate binding. Biophys. J. 103, 1790–1801. doi: 10.1016/j.bpj.2012.09.009, PMID: 23083723 PMC3475387

[ref21] Fisher J. Mobashery S. (2009). Three decades of the class A beta-lactamase acyl-enzyme. CPPS 10, 401–407. doi: 10.2174/138920309789351967, PMID: 19538154 PMC6902449

[ref22] Fisher J. F. Mobashery S. (2016). β-Lactam resistance mechanisms: gram-positive bacteria and mycobacterium tuberculosis. Cold Spring Harb. Perspect. Med. 6:a025221. doi: 10.1101/cshperspect.a025221 27091943 PMC4852796

[ref23] Fonseca F. Chudyk E. I. van der Kamp M. W. Correia A. Mulholland A. J. Spencer J. (2012). The basis for carbapenem hydrolysis by class A β-lactamases: a combined investigation using crystallography and simulations. J. Am. Chem. Soc. 134, 18275–18285. doi: 10.1021/ja304460j, PMID: 23030300

[ref24] Galdadas I. Lovera S. Pérez-Hernández G. Barnes M. D. Healy J. Afsharikho H.. (2018). Defining the architecture of KPC-2 Carbapenemase: identifying allosteric networks to fight antibiotics resistance. Sci. Rep. 8:12916. doi: 10.1038/s41598-018-31176-0 30150677 PMC6110804

[ref25] Galdadas I. Qu S. Oliveira A. S. F. Olehnovics E. Mack A. R. Mojica M. F.. (2021). Allosteric communication in class A β-lactamases occurs *via* cooperative coupling of loop dynamics. elife 10:e66567. doi: 10.7554/eLife.66567 33755013 PMC8060031

[ref26] Gobeil S. M. C. Ebert M. C. C. J. C. Park J. Gagné D. Doucet N. Berghuis A. M.. (2019). The structural dynamics of engineered β-lactamases vary broadly on three timescales yet sustain native function. Sci. Rep. 9:6656. doi: 10.1038/s41598-019-42866-8 31040324 PMC6491436

[ref27] Grigorenko V. G. Andreeva I. P. Rubtsova M. Y. Deygen I. M. Antipin R. L. Majouga A. G.. (2017). Novel non-β-lactam inhibitor of β-lactamase TEM-171 based on acylated phenoxyaniline. Biochimie 132, 45–53. doi: 10.1016/j.biochi.2016.10.011, PMID: 27771370

[ref28] Haidar G. Clancy C. J. Shields R. K. Hao B. Cheng S. Nguyen M. H. (2017). Mutations in blaKPC-3 That confer Ceftazidime-avibactam resistance encode novel KPC-3 variants That function as extended-Spectrum β-lactamases. Antimicrob. Agents Chemother. 61:e0253-16. doi: 10.1128/AAC.02534-16, PMID: 28223379 PMC5404534

[ref29] Harris P. N. A. Tambyah P. A. Lye D. C. Mo Y. Lee T. H. Yilmaz M.. (2018). Effect of piperacillin-Tazobactam vs Meropenem on 30-day mortality for patients With *E coli* or *Klebsiella pneumoniae* bloodstream infection and ceftriaxone resistance: a randomized clinical trial. JAMA 320, 984–994. doi: 10.1001/jama.2018.12163, PMID: 30208454 PMC6143100

[ref30] Hart K. M. Ho C. M. W. Dutta S. Gross M. L. Bowman G. R. (2016). Modelling proteins’ hidden conformations to predict antibiotic resistance. Nat. Commun. 7:12965. doi: 10.1038/ncomms12965 27708258 PMC5477488

[ref31] Harvey M. J. Giupponi G. Fabritiis G. D. (2009). ACEMD: accelerating biomolecular dynamics in the microsecond time scale. J. Chem. Theory Comput. 5, 1632–1639. doi: 10.1021/ct9000685, PMID: 26609855

[ref32] Heo Y.-A. (2021). Imipenem/Cilastatin/Relebactam: A review in gram-negative bacterial infections. Drugs 81, 377–388. doi: 10.1007/s40265-021-01471-8, PMID: 33630278 PMC7905759

[ref33] Horn J. R. Shoichet B. K. (2004). Allosteric inhibition Through Core disruption. J. Mol. Biol. 336, 1283–1291. doi: 10.1016/j.jmb.2003.12.068, PMID: 15037085

[ref34] Humphrey W. Dalke A. Schulten K. (1996). VMD: visual molecular dynamics. J. Mol. Graph. 14, 27–28. doi: 10.1016/0263-7855(96)00018-5 8744570

[ref35] Kalp M. Carey P. R. (2008). Carbapenems and SHV-1 β-lactamase form different acyl-enzyme populations in crystals and solution. Biochemistry 47, 11830–11837. doi: 10.1021/bi800833u, PMID: 18922024 PMC2656688

[ref36] Ke W. Bethel C. R. Thomson J. M. Bonomo R. A. van den Akker F. (2007). Crystal structure of KPC-2: insights into carbapenemase activity in class A β-lactamases. Biochemistry 46, 5732–5740. doi: 10.1021/bi700300u, PMID: 17441734 PMC2596071

[ref37] Kingma D. P. Welling M. (2014). Auto-Encoding Variational Bayes. arXiv:1312.6114 [cs, stat]. Available at: http://arxiv.org/abs/1312.6114 (Accessed 6 May, 2021).

[ref38] Krajnc A. Lang P. A. Panduwawala T. D. Brem J. Schofield C. J. (2019). Will morphing boron-based inhibitors beat the β-lactamases? Curr. Opin. Chem. Biol. 50, 101–110. doi: 10.1016/j.cbpa.2019.03.001, PMID: 31004962 PMC6591701

[ref39] Lagacé-Wiens P. Walkty A. Karlowsky J. A. (2014). Ceftazidime-avibactam: an evidence-based review of its pharmacology and potential use in the treatment of gram-negative bacterial infections. Core Evid 9, 13–25. doi: 10.2147/CE.S40698, PMID: 24493994 PMC3908787

[ref40] Langan P. S. Vandavasi V. G. Cooper S. J. Weiss K. L. Ginell S. L. Parks J. M.. (2018). Substrate binding induces conformational changes in a class A β-lactamase That prime it for catalysis. ACS Catal. 8, 2428–2437. doi: 10.1021/acscatal.7b04114

[ref41] Laskowski R. A. Gerick F. Thornton J. M. (2009). The structural basis of allosteric regulation in proteins. FEBS Lett. 583, 1692–1698. doi: 10.1016/j.febslet.2009.03.019, PMID: 19303011

[ref42] Le Guilloux V. Schmidtke P. Tuffery P. (2009). Fpocket: An open source platform for ligand pocket detection. BMC Bioinformatics 10:168. doi: 10.1186/1471-2105-10-168, PMID: 19486540 PMC2700099

[ref43] Leavitt A. Navon-Venezia S. Chmelnitsky I. Schwaber M. J. Carmeli Y. (2007). Emergence of KPC-2 and KPC-3 in Carbapenem-resistant *Klebsiella pneumoniae* strains in an Israeli hospital. Antimicrob. Agents Chemother. 51, 3026–3029. doi: 10.1128/AAC.00299-07, PMID: 17562800 PMC1932543

[ref44] Maier J. A. Martinez C. Kasavajhala K. Wickstrom L. Hauser K. E. Simmerling C. (2015). ff14SB: improving the accuracy of protein side chain and backbone parameters from ff99SB. J. Chem. Theory Comput. 11, 3696–3713. doi: 10.1021/acs.jctc.5b00255, PMID: 26574453 PMC4821407

[ref45] Majiduddin F. K. Palzkill T. (2003). Amino acid sequence requirements at residues 69 and 238 for the SME-1 β-lactamase to confer resistance to β-lactam antibiotics. Antimicrob. Agents Chemother. 47, 1062–1067. doi: 10.1128/AAC.47.3.1062-1067.2003, PMID: 12604542 PMC149323

[ref46] Martínez L. (2015). Automatic identification of Mobile and rigid substructures in molecular dynamics simulations and fractional structural fluctuation analysis. PLoS One 10:e0119264. doi: 10.1371/journal.pone.0119264, PMID: 25816325 PMC4376797

[ref47] Martínez-Rosell G. Giorgino T. De Fabritiis G. (2017). PlayMolecule ProteinPrepare: A web application for protein preparation for molecular dynamics simulations. J. Chem. Inf. Model. 57, 1511–1516. doi: 10.1021/acs.jcim.7b00190 28594549

[ref48] McGibbon R. T. Beauchamp K. A. Harrigan M. P. Klein C. Swails J. M. Hernández C. X.. (2015). MDTraj: A modern open library for the analysis of molecular dynamics trajectories. Biophys. J. 109, 1528–1532. doi: 10.1016/j.bpj.2015.08.015, PMID: 26488642 PMC4623899

[ref49] Mehta S. C. Rice K. Palzkill T. (2015). Natural variants of the KPC-2 Carbapenemase have evolved increased catalytic efficiency for Ceftazidime hydrolysis at the cost of enzyme stability. PLoS Pathog. 11:e1004949. doi: 10.1371/journal.ppat.1004949, PMID: 26030609 PMC4452179

[ref50] Meneksedag D. Dogan A. Kanlikilicer P. Ozkirimli E. (2013). Communication between the active site and the allosteric site in class A beta-lactamases. Comput. Biol. Chem. 43, 1–10. doi: 10.1016/j.compbiolchem.2012.12.002, PMID: 23314151

[ref51] Meroueh S. O. Roblin P. Golemi D. Maveyraud L. Vakulenko S. B. Zhang Y.. (2002). Molecular dynamics at the root of expansion of function in the M69L inhibitor-resistant TEM β-lactamase from *Escherichia coli*. J. Am. Chem. Soc. 124, 9422–9430. doi: 10.1021/ja026547q, PMID: 12167037

[ref52] Motlagh H. N. Wrabl J. O. Li J. Hilser V. J. (2014). The ensemble nature of allostery. Nature 508, 331–339. doi: 10.1038/nature13001, PMID: 24740064 PMC4224315

[ref53] Naas T. Dortet L. Iorga B. I. (2016). Structural and functional aspects of class A Carbapenemases. Curr. Drug Targets 17, 1006–1028. doi: 10.2174/1389450117666160310144501 26960341 PMC5405625

[ref54] Palzkill T. (2018). Structural and mechanistic basis for extended-Spectrum drug-resistance mutations in altering the specificity of TEM, CTX-M, and KPC β-lactamases. Front. Mol. Biosci. 5:16. doi: 10.3389/fmolb.2018.00016 29527530 PMC5829062

[ref55] Papp-Wallace K. M. Mack A. R. Taracila M. A. Bonomo R. A. (2020). Resistance to novel β-lactam–β-lactamase inhibitor combinations. Infect. Dis. Clin. N. Am. 34, 773–819. doi: 10.1016/j.idc.2020.05.001, PMID: 33011051 PMC7609624

[ref56] Papp-Wallace K. M. Taracila M. Wallace C. J. Hujer K. M. Bethel C. R. Hornick J. M.. (2010). Elucidating the role of Trp105 in the KPC-2 β-lactamase: The role of Trp105 in the KPC-2 β-lactamase. Protein Sci. 19, 1714–1727. doi: 10.1002/pro.454, PMID: 20662006 PMC2975135

[ref57] Partridge S. R. Kwong S. M. Firth N. Jensen S. O. (2018). Mobile genetic elements associated with antimicrobial resistance. Clin. Microbiol. Rev. 31:e00088-17. doi: 10.1128/CMR.00088-17 30068738 PMC6148190

[ref58] Pemberton O. A. Noor R. E. Vasantha Kumar M. V. Sanishvili R. Kemp M. T. Kearns F. L.. (2020). Mechanism of proton transfer in class A β-lactamase catalysis and inhibition by avibactam. PNAS 117, 5818–5825. doi: 10.1073/pnas.1922203117 32123084 PMC7084157

[ref59] Pemberton O. A. Zhang X. Chen Y. (2017). Molecular basis of substrate recognition and product release by the *Klebsiella pneumoniae* Carbapenemase (KPC-2). J. Med. Chem. 60, 3525–3530. doi: 10.1021/acs.jmedchem.7b00158, PMID: 28388065 PMC5506774

[ref60] Roe D. R. Cheatham T. E. (2013). PTRAJ and CPPTRAJ: software for processing and analysis of molecular dynamics trajectory data. J. Chem. Theory Comput. 9, 3084–3095. doi: 10.1021/ct400341p, PMID: 26583988

[ref61] Romero R. Ramanathan A. Yuen T. Bhowmik D. Mathew M. Munshi L. B.. (2019). Mechanism of glucocerebrosidase activation and dysfunction in Gaucher disease unraveled by molecular dynamics and deep learning. Proc. Natl. Acad. Sci. U. S. A. 116, 5086–5095. doi: 10.1073/pnas.1818411116, PMID: 30808805 PMC6421449

[ref62] Scherer M. K. Trendelkamp-Schroer B. Paul F. Pérez-Hernández G. Hoffmann M. Plattner N.. (2015). PyEMMA 2: a software package for estimation, validation, and analysis of Markov models. J. Chem. Theory Comput. 11, 5525–5542. doi: 10.1021/acs.jctc.5b00743, PMID: 26574340

[ref63] Shields R. K. Chen L. Cheng S. Chavda K. D. Press E. G. Snyder A.. (2017). Emergence of Ceftazidime-avibactam resistance due to plasmid-borne blaKPC-3 mutations during treatment of Carbapenem-resistant *Klebsiella pneumoniae* infections. Antimicrob. Agents Chemother. 61:e02097-16. doi: 10.1128/AAC.02097-16, PMID: 28031201 PMC5328542

[ref64] Shimamura T. Ibuka A. Fushinobu S. Wakagi T. Ishiguro M. Ishii Y.. (2002). Acyl-intermediate structures of the extended-spectrum class A β-lactamase, Toho-1, in complex with Cefotaxime, Cephalothin, and Benzylpenicillin. J. Biol. Chem. 277, 46601–46608. doi: 10.1074/jbc.M207884200 12221102

[ref65] Stoesser N. Sheppard A. E. Peirano G. Anson L. W. Pankhurst L. Sebra R.. (2017). Genomic epidemiology of global *Klebsiella pneumoniae* carbapenemase (KPC)-producing *Escherichia coli*. Sci. Rep. 7:5917. doi: 10.1038/s41598-017-06256-2 28725045 PMC5517641

[ref66] Tomasello G. Armenia I. Molla G. (2020). The protein imager: a full-featured online molecular viewer interface with server-side HQ-rendering capabilities. Bioinformatics 36, 2909–2911. doi: 10.1093/bioinformatics/btaa009, PMID: 31930403

[ref67] Tooke C. L. Hinchliffe P. Bragginton E. C. Colenso C. K. Hirvonen V. H. A. Takebayashi Y.. (2019). β-Lactamases and β-lactamase inhibitors in the 21st century. J. Mol. Biol. 431, 3472–3500. doi: 10.1016/j.jmb.2019.04.002, PMID: 30959050 PMC6723624

[ref68] Tooke C. L. Hinchliffe P. Krajnc A. Mulholland A. J. Brem J. Schofield C. J.. (2020). Cyclic boronates as versatile scaffolds for KPC-2 β-lactamase inhibition. RSC Med. Chem. 11, 491–496. doi: 10.1039/C9MD00557A, PMID: 33479650 PMC7536818

[ref69] Towns J. Cockerill T. Dahan M. Foster I. Gaither K. Grimshaw A.. (2014). XSEDE: accelerating scientific discovery. Comput. Sci. Eng. 16, 62–74. doi: 10.1109/MCSE.2014.80

[ref70] Wang X. Minasov G. Shoichet B. K. (2002). The structural bases of antibiotic resistance in the clinically derived mutant beta-lactamases TEM-30, TEM-32, and TEM-34. J. Biol. Chem. 277, 32149–32156. doi: 10.1074/jbc.M204212200, PMID: 12058046

[ref71] Wells B. A. Chaffee A. L. (2015). Ewald summation for molecular simulations. J. Chem. Theory Comput. 11, 3684–3695. doi: 10.1021/acs.jctc.5b00093, PMID: 26574452

[ref72] Zafaralla G. Mobashery S. (1992). Facilitation of the. DELTA.2.Fwdarw. DELTA.1 pyrroline tautomerization of carbapenem antibiotics by the highly conserved arginine-244 of class A. Beta.-lactamases during the course of turnover. J. Am. Chem. Soc. 114, 1505–1506. doi: 10.1021/ja00030a070

