# Supplementary material for: The Role of Hydrophobic Nodes in the Dynamics of Class A β-Lactamases
Source: Front Microbiol. 2021 Sep 21;12:720991. doi: 10.3389/fmicb.2021.720991 (PMC8490755; doi:10.3389/fmicb.2021.720991)
Supplement: Supplementary file 1 [file Data_Sheet_1.pdf]

Supplementary Information

The role of hydrophobic nodes in the dynamics of class A β-lactamases

Edgar Olehnovics, Junqi Yin, Adrià Pérez, Gianni De Fabritiis, Robert A Bonomo, Debsindhu Bhowmik and Shozeb Haider*

* Corresponding author: Shozeb Haider
Email: Shozeb.haider@ucl.ac.uk
ORCID: Shozeb Haider: 0000-0003-2650-2925

Table S1: Hydrophobic nodes

Nodes	KPC-2	SME-1	SHV-1	TEM-1
**α2**	GFLAAAVLA<br>74-82	GFLAAAVLE<br>74-82	VVLCGAVLA<br>74-82	VVLCGAVLS<br>74-82
**α3**	ALV<br>101-103	DLE<br>101-103	DLV<br>101-103	DLV<br>101-103
**α4**	SPI<br>106-108	SPI<br>106-108	SPV<br>106-108	SPV<br>106-108
**α5**	AAAV<br>124-127	SAAL<br>124-127	AAAI<br>124-127	SAAI<br>124-127
**α6**	AAA<br>133-135	GAT<br>133-135	SAA<br>133-135	TAA<br>133-135
**α6**	LLL<br>137-139	IIM<br>137-139	LLL<br>137-139	LLL<br>137-139
**α9**	AVT<br>185-187	AVA<br>185-187	SMA<br>185-187	AMA<br>185-187
**α10**	ALAA<br>198-201	VLNA<br>198-201	RLSA<br>198-201	LLTL<br>198-201
**α11**	AAV<br>223-225	ASV<br>223-225	SVL<br>223-225	SAL<br>223-225
**β7**	AVG<br>230-232	VVG<br>230-232	FIA<br>230-232	FIA<br>230-232
**β8**	AVV<br>248-250	AVI<br>248-250	ALL<br>248-250	AAL<br>248-250
**β9**	VLA<br>260-262	AVS<br>260-262	AVV<br>260-262	AVV<br>260-262
**α12**	AAAA<br>280-283	AEAS<br>280-283	AGIG<br>280-283	AEIG<br>280-283

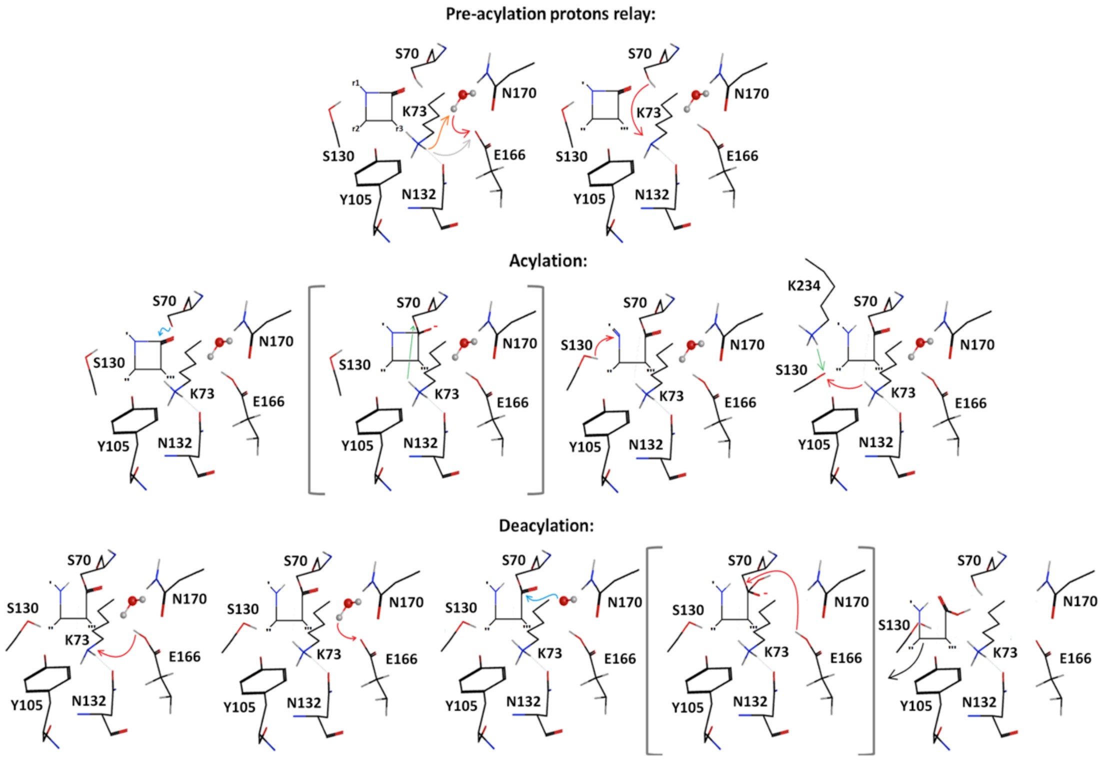

Figure S1 | Catalytic mechanism of β-lactam hydrolysis in TEM-1. In class-A β-lactamase at physiological pH, **K73** remains protonated and **E166** deprotonated. Entry of an appropriately positioned β-lactam in the active site forms a reversible Michaelis complex, where stabilization of the carbonyl-group of the β-lactam ring adjacent to S70 alters the electrostatic free energy landscape in neighbouring residues, triggering a rapid relay of proton transfer events (red and orange arrows) involving **K73** and **E166**; resulting in a deprotonated **S70**(Oγ). This is followed by the acylation reactions which is initiated by a nucleophilic attack on the carbonyl carbon by the lone pair of Oγ electrons (cyan arrow), forming the first unstable acyl-enzyme tetrahedral covalent intermediate, which may be transiently stabilised by a low barrier hydrogen bond (HB) from **K73** (dashed green arrow). This HB also lowers activation energy prior to tetrahedral intermediate formation (Hata et al., 2000). High-energy tetrahedral intermediate energetically resolves via the collapse of the β-lactam endocyclic amide bond, where a proton is simultaneously donated to the valent nitrogen from the nearby **S130** side chain, forming an acyl-enzyme adduct (first stable transition intermediate). This covalent intermediate may be long-lived depending on the chemistry of the β-lactam and the topology of the active site. Deacylation reaction proceeds by a proton transfer from **K73** to **S130**, where **K73** is restored by accepting a proton from **E166**. If the hydrolytic water molecule is stable and positioned <3.5Å from the electrophilic carbonyl carbon, the water becomes activated by **E166**, forming a hydroxide ion which immediately hydrolyses the carbonyl carbon forming the second unstable tetrahedral intermediate. The hydrolysed β-lactam is released when the acyl-enzyme bond collapses, which may be energetically favourable as soon as **E166**(ε2) proton approaches near to **S70**(Oγ). The reacted β-lactam product, which is released from the active site no longer contains intact β-lactam ring, leaving it unreactive against PBP. (Pemberton et al., 2020; Langan et al., 2018)

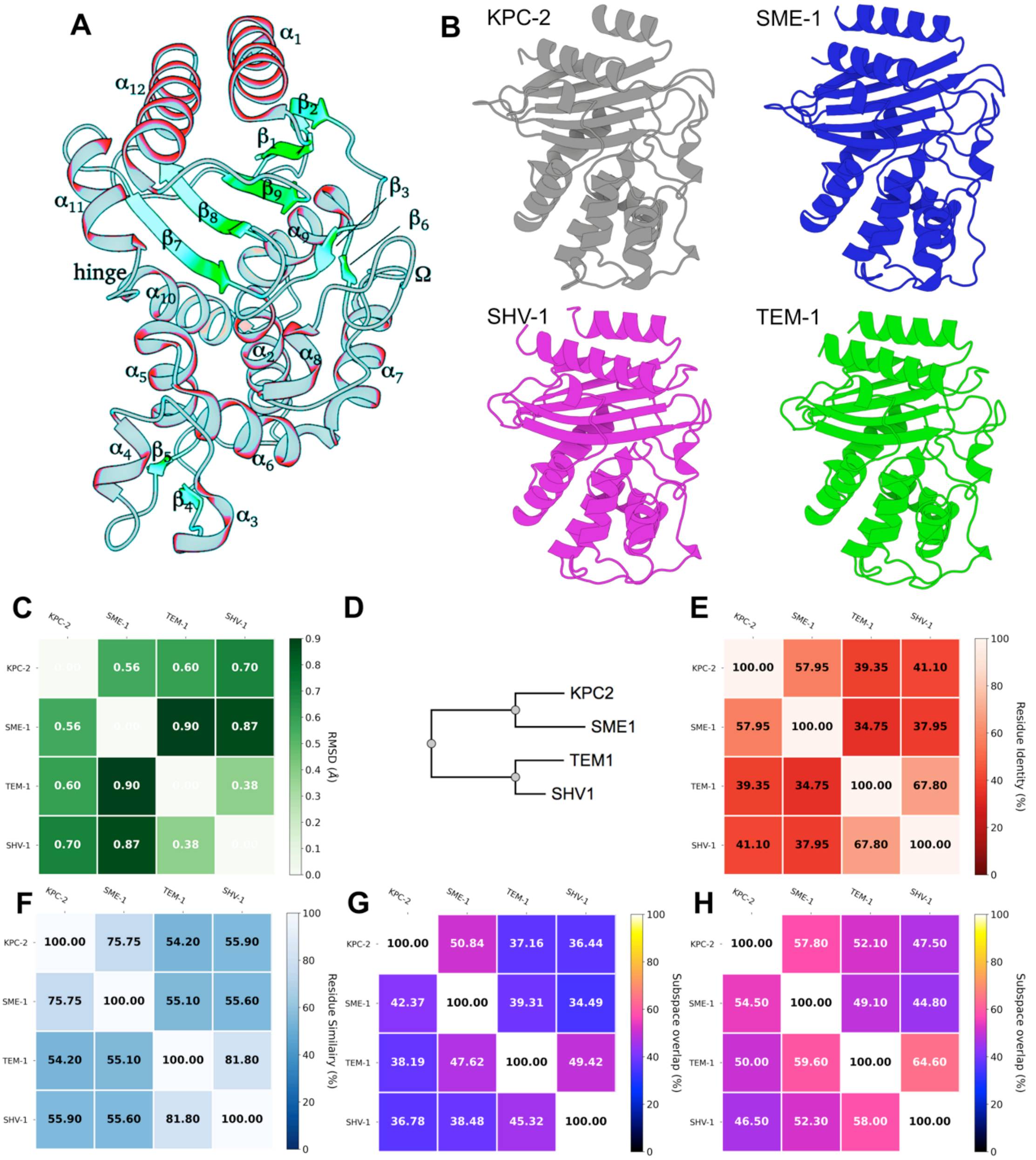

Figure S2 | Sequence and structural alignments. (A) The class A β-lactamase structural nomenclature; (B) Crystal structures of KPC-2 (PDB id 2OV5), SME-1 (PDB id 1DY6), SHV-1 (PDB id 3N4I) and TEM-1 (PDB id 1XPB); (C) Cα RMSD of the structures; (D) Residue identity (% of residues with exact match at given position); (E) Residue similarity (% of residues with similar side chain chemistry); (F) Phylogenetic similarities (calculated at phylogeny.fr); (G) Dynamic subspace overlaps (%) based on structurally analogous Cα atoms (bottom triangle: Cα coordinates, top triangle: Cα contact maps); (H) Dynamic subspace overlaps (%) based on Cα atoms of the hydrophobic node residues (bottom triangle: Cα coordinates, top triangle: Cα contact maps).

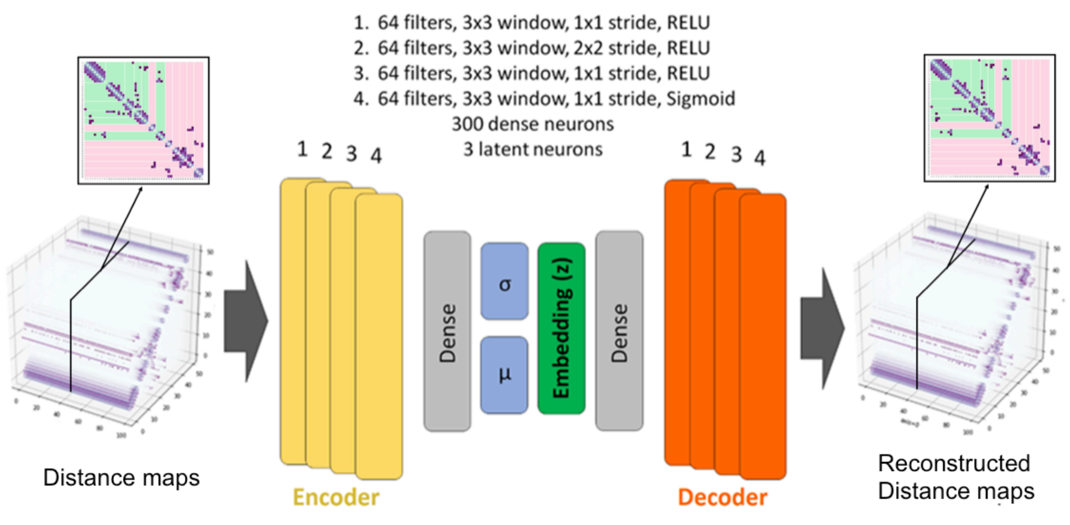

Figure S3. Deep Learning Implementation. An overview of the CVAE architecture where distance maps used for training and validating the CVAE model comprise Cα distances (<8 Å) between the residues, which are members of the α and β hydrophobic networks. The neural network architecture, which was adopted for this image clustering, is illustrated. Convolutional layers of the encoder and decoder are coloured yellow and orange, respectively. The architectures used 24,266,091 trainable parameters in total. The linear latent layers are coloured blue and green, with μ and σ representing the learned parameters (mean and standard deviation), which parameterise the Gaussian distribution centred at the origin (z).

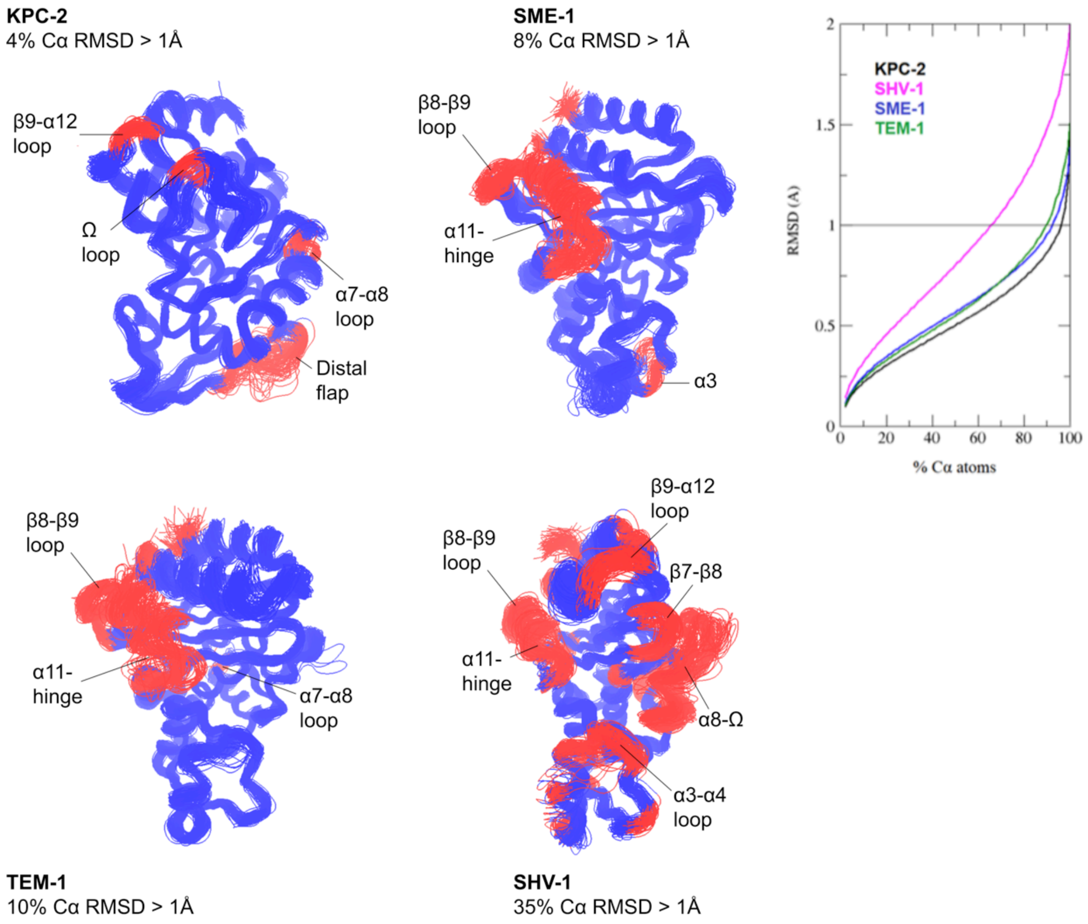

Figure S4 | MDlovofit results showing showing regions of the backbone where regional Cα-RMSD is >1Å. Percentage of % Cα atoms with RMSD >1Å were 4, 8, 10 and 35 % in KPC-2, SME-1, TEM-1 and SHV-1 respectively. Highly dynamic regions are coloured red.

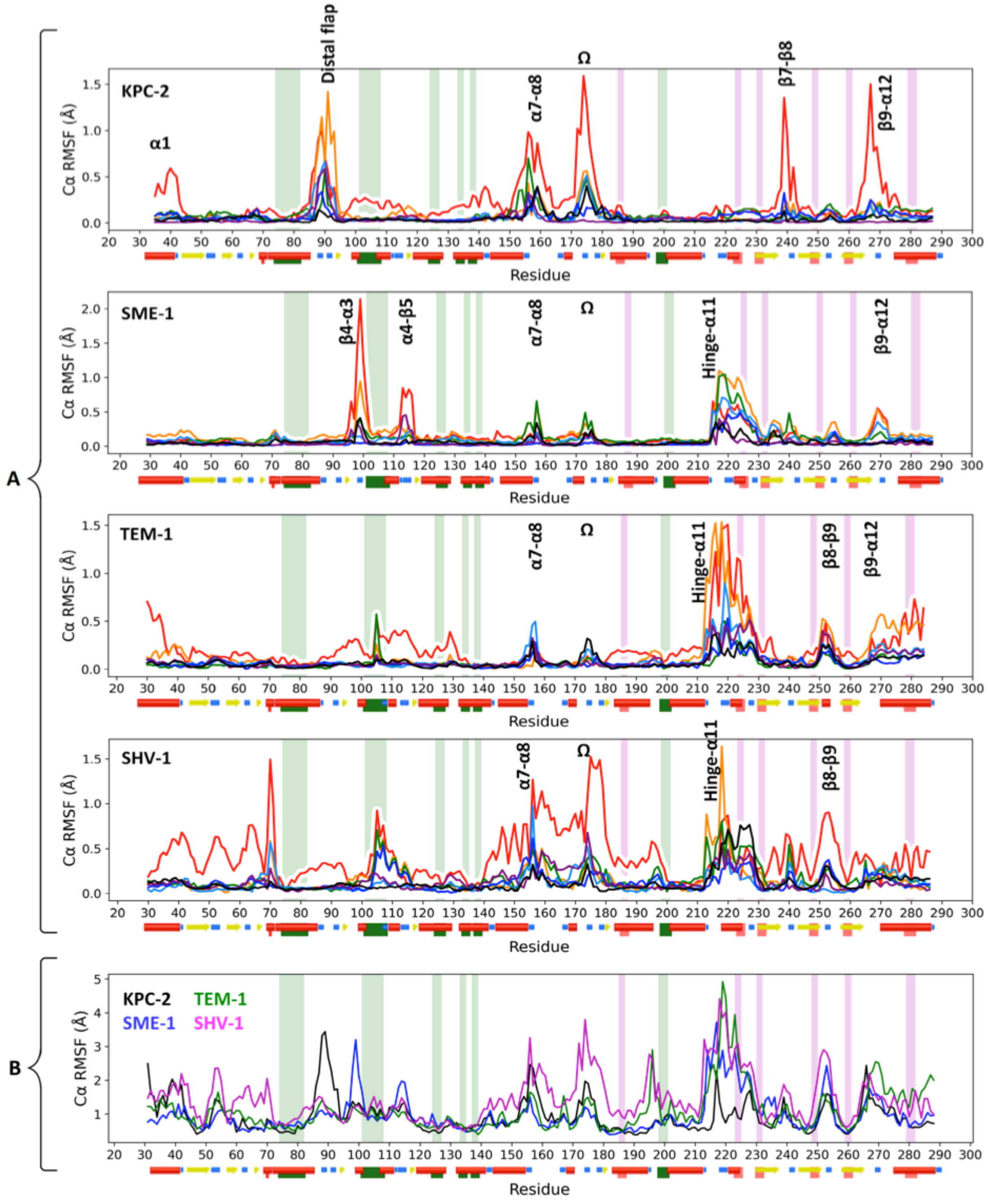

Figure S5 | (**A**) Root Mean Squared Fluctuation (RMSF) plots generated after independently filtering mean-free Cα trajectories one time-step through top 7 rank-1 linear Koopman matrices, illustrating magnitudes Cα displacements separately incurred by top 7 slowest liner dynamic modes, which were estimated using time lagged independent component analysis at lag-time of 0.5ns in each system. The colours follow from red to black, with red referred to as "IC1" in text, and orange as "IC2", etc. (**B**) Conventional Cα-RMSF in the four enzymes, with residues on the x-axis aligned exactly as indicated by multi-sequence alignment. All sequences were aligned to KPC-2 numbering.

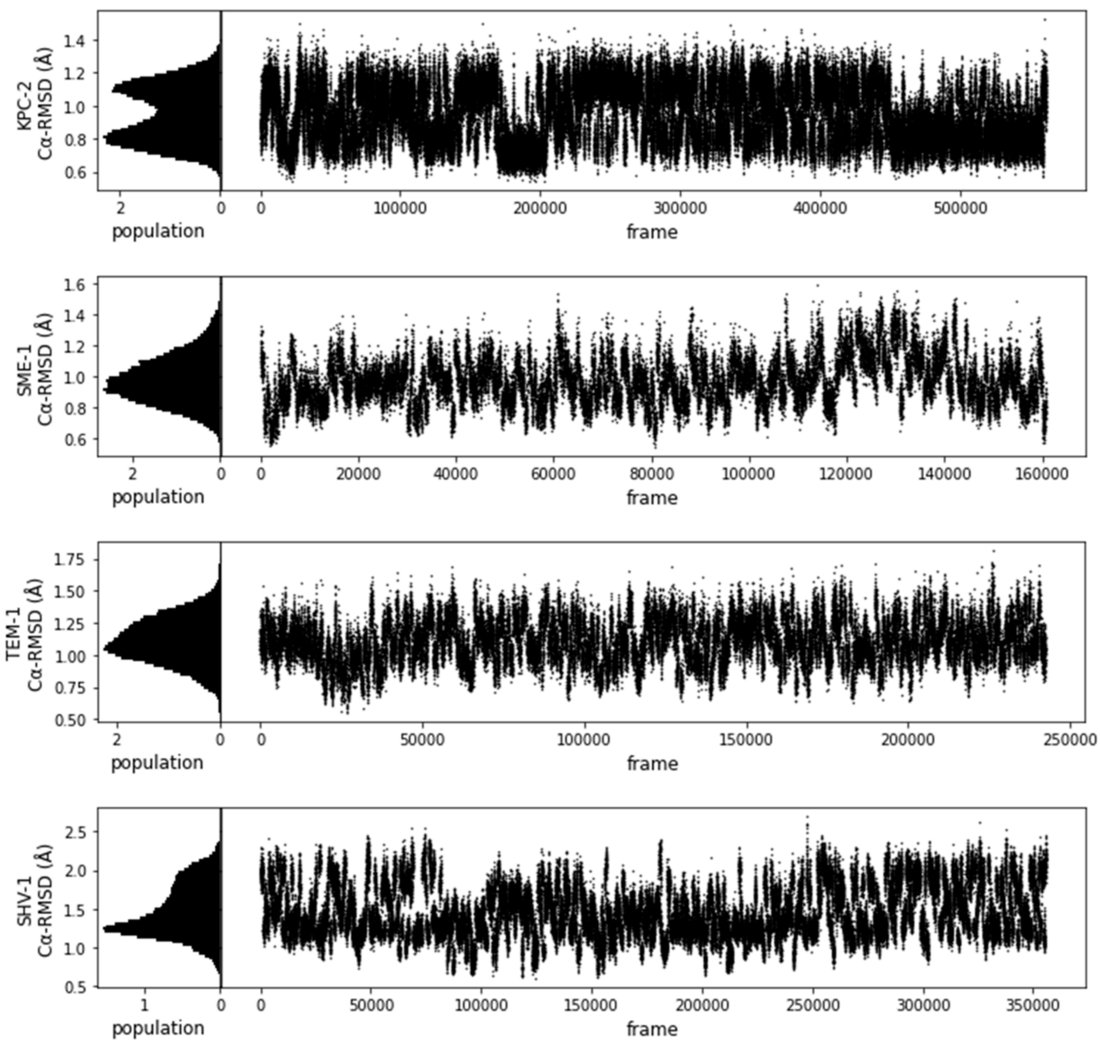

Figure S6 | Root Mean Squared Deviation (RMSD) of Cα-atoms relative to crystal structure. Timestep between frames is 0.1ns.

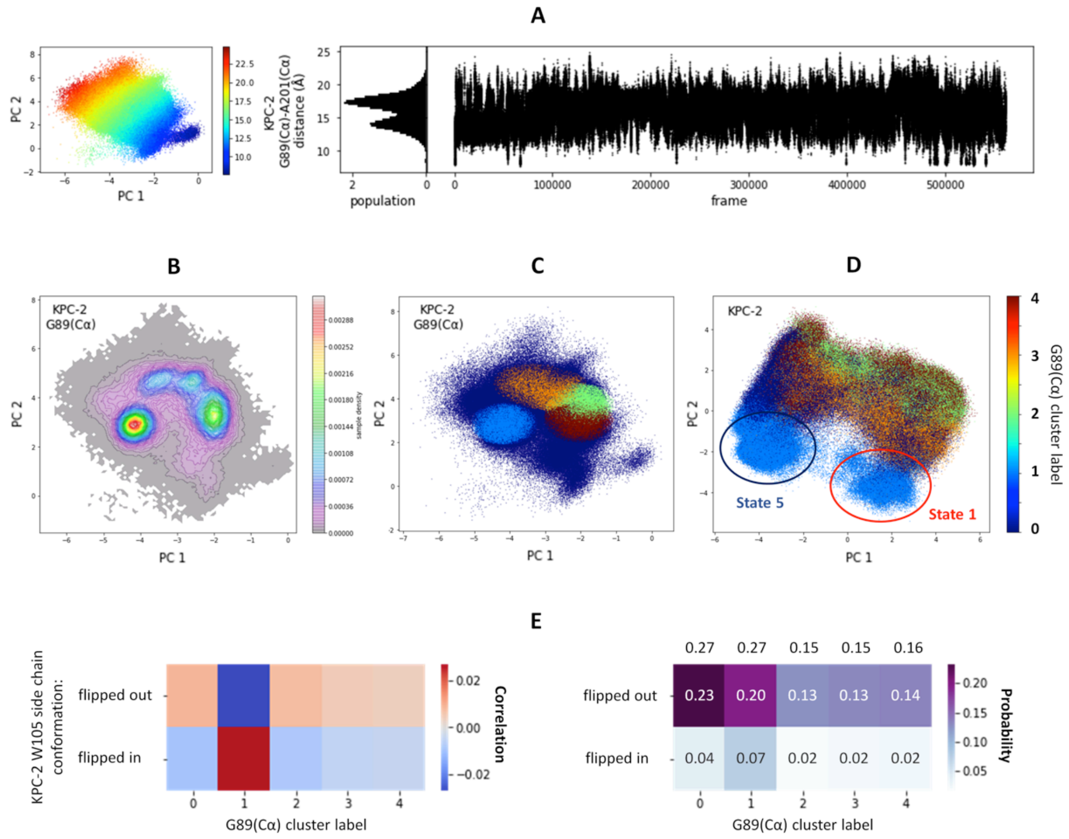

Figure S7 | Distal flap dynamics in KPC-2. The metastable 'open-flap' conformation of the distal flap (as observed in MSM states 1 and 5) is correlated positively with 'flipped-in' conformation in W105 side-chain. **(A)** Distance between G89(Cα) and A201(Cα) atoms was chosen as a metric of distal flap conformation ('open' when this distance is higher and 'closed' when this distance is lower); **(B)** Stationary distribution of the G89(Cα) atom visualised using top two eigenvectors, indicating that the 'open' conformation is the global FE minimum of the distal flap; **(C)** Coordinates of G89(Cα) atom clustered by k-means into 5 discrete states. Open-flap is coloured light blue, closed-flap is red/green, and the dark blue cloud represents lack of metastability in the distal flap; **(D)** KPC-2 system-wide PCA projection showing that the highly stable 'open-flap' conformation of the distal flap occurs in MSM states 1 and 5; **(E)** Cross-correlation matrix and joined probability histogram showing that the 'open' conformation of the distal flap is positively coupled to W105 sidechain being flipped-in. This correlation, which is weak in magnitude, may only be relevant in MSM states 1 and 5. The likely allosteric pathway of this long-range influence is via the hydrophobic network of the α-subdomain, as indicated by information plot in Figure S8. W105 loop receives a combination of multimodal allosteric inputs from dynamics of other loops. These inter-loop communication are relayed via the (hydrophobic) core.

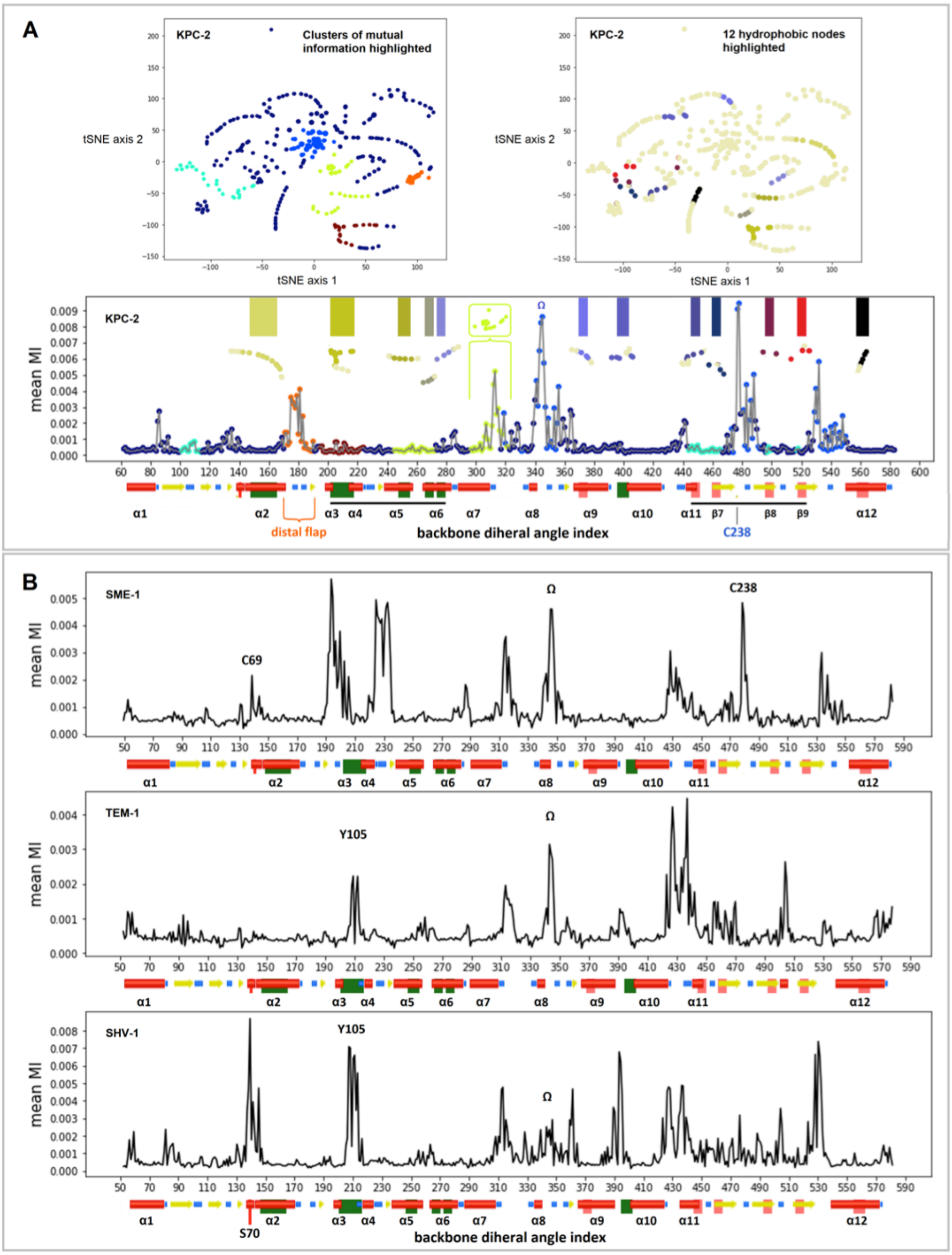

Figure S8 | Mean normalized mutual information (nMI) plots based on backbone dihedral angles. In **A** (KPC-2), a further step was taken to embed the nMI distance matrix (1-nMI) onto a 2D plot, where each point represents a dihedral angle. This embedding illustrates approximate allosteric signaling pathways, showing three important 'sources' (peaks) of global information (the Ω-loop: in close coupling to C238 region (light blue), the α7-α8 loop (lime colored box), and the distal flap (orange)). Internal coupling within the two sets of hydrophobic nodes ({α3-α4, α5, α6}, {α11, β7, β8, β9}) is shown. This behaviour can be readily supported alternatively by normalised transfer entropy (with reshuffling), and in both KPC-2 and SME-1. In **A** and **B**, there are notable differences in magnitudes of average global nMI described by the loops. These averages can be considered, given the consistent use of hyper-parameter (10 bins per angle).

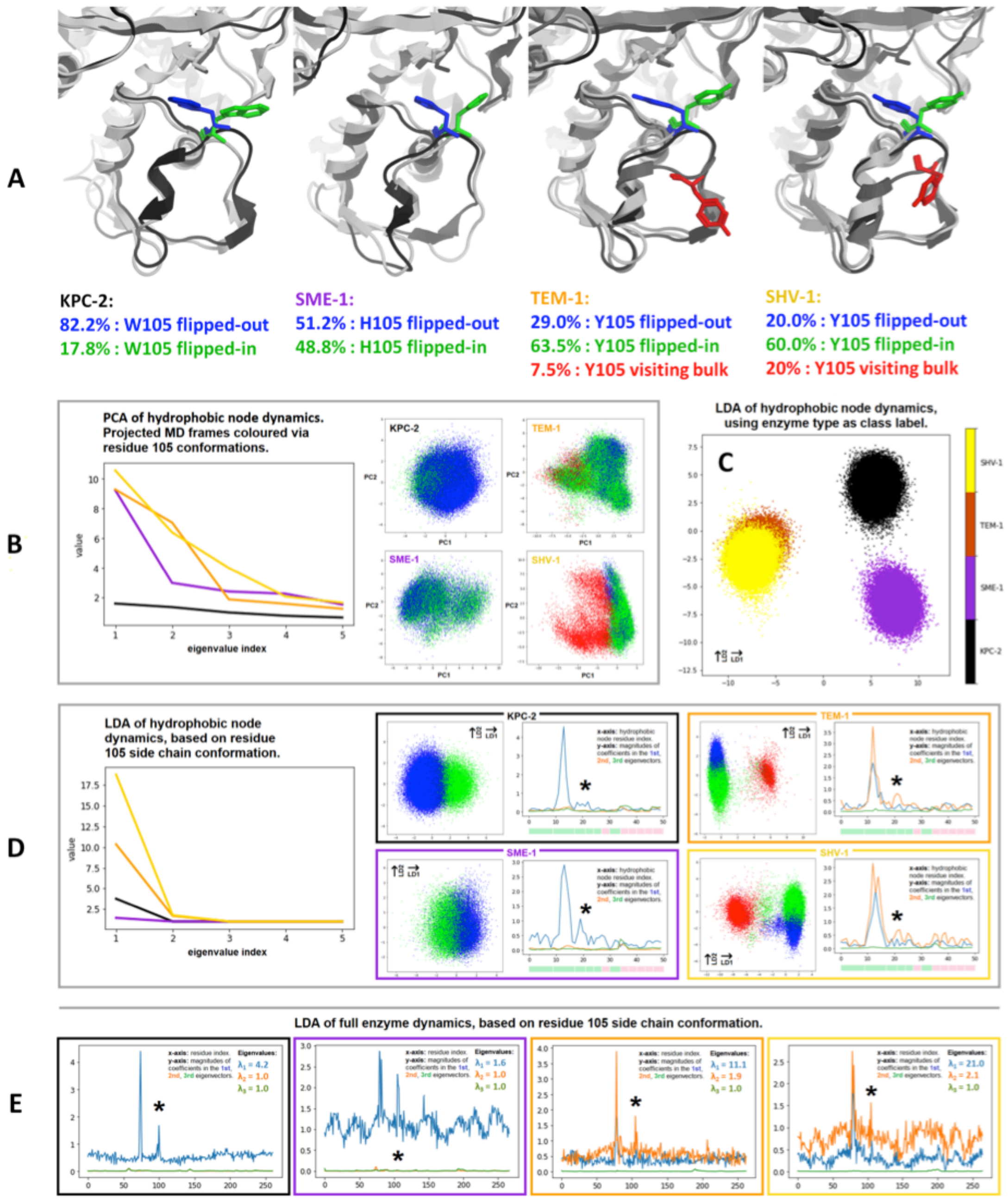

Figure S9 | (**A**) Prevalence of the residue 105 side chain conformations from adaptively sampled MD trajectories in the four enzymes. (**B**) PCA of hydrophobic node Cα dynamics, showing relatively weak unsupervised state separation with respect to residue 105 side chain conformations. (**C**) LDA resolving Cα dynamics of the hydrophobic networks in the four systems. TEM-1 and SHV-1 are resolved by third eigenvector LD3 (not shown). (**D**) Linear Discriminant Analysis (LDA) resolving the Cα dynamics of the hydrophobic network in each system w.r.t. residue 105 side chain conformations. SME-1 stands out with lowest top eigenvalues and noisy state separation. α5-α6 region is highlighted with a *, while the tallest peak is the 105(Cα) atom and the α3-α4 loop. (**E**) LDA resolving the Cα dynamics of the complete enzymes w.r.t. residue 105 side chain conformations. α5-α6 loop (*) stand out as important in relation to ‘flipped-in’ vs. ‘flipped-out’ states.

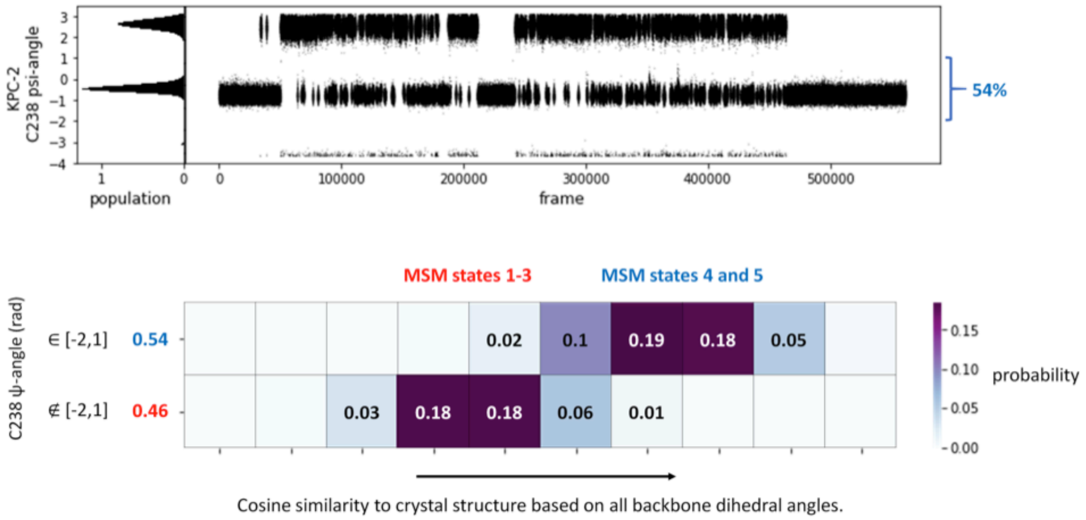

Figure S10 | Concatenated trajectories of KPC-2 C238 ψ-angle show clear separation into two metastable states. These two states are directly correlated with the global double well stationary distribution in KPC-2.

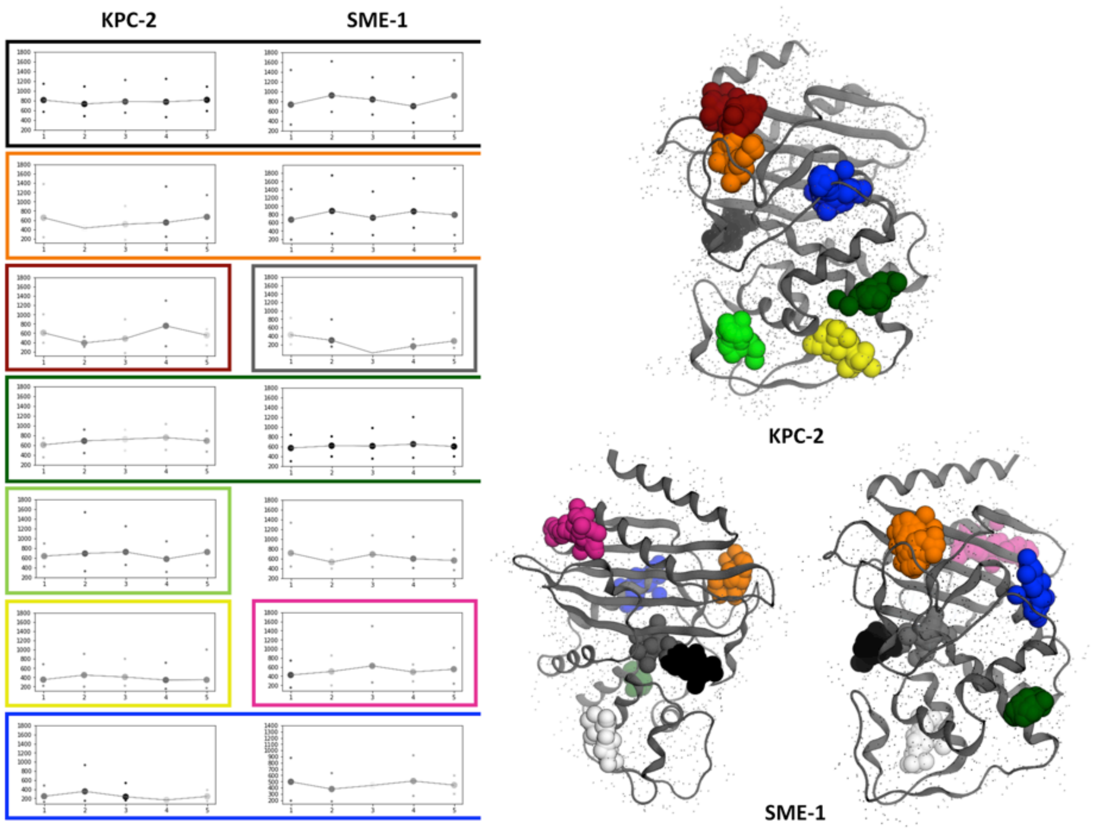

Figure S11 | KPC-2 and SME-1 average pocket volumes (Å^3) calculated in the five metastable states. The volumes were calculated with 95% CI (based on 20 measurements or less). The number of measurements is proportional to colour intensity of the circles on the plots. The x-axis represent metastable PCCA states in which a pocket at a given location was found, and the darkness (colour intensity) of the points illustrates prevalence of the given pocket at the given metastable conformation. Pockets in cartoons are illustrated using average coordinates of fpocket vertices.

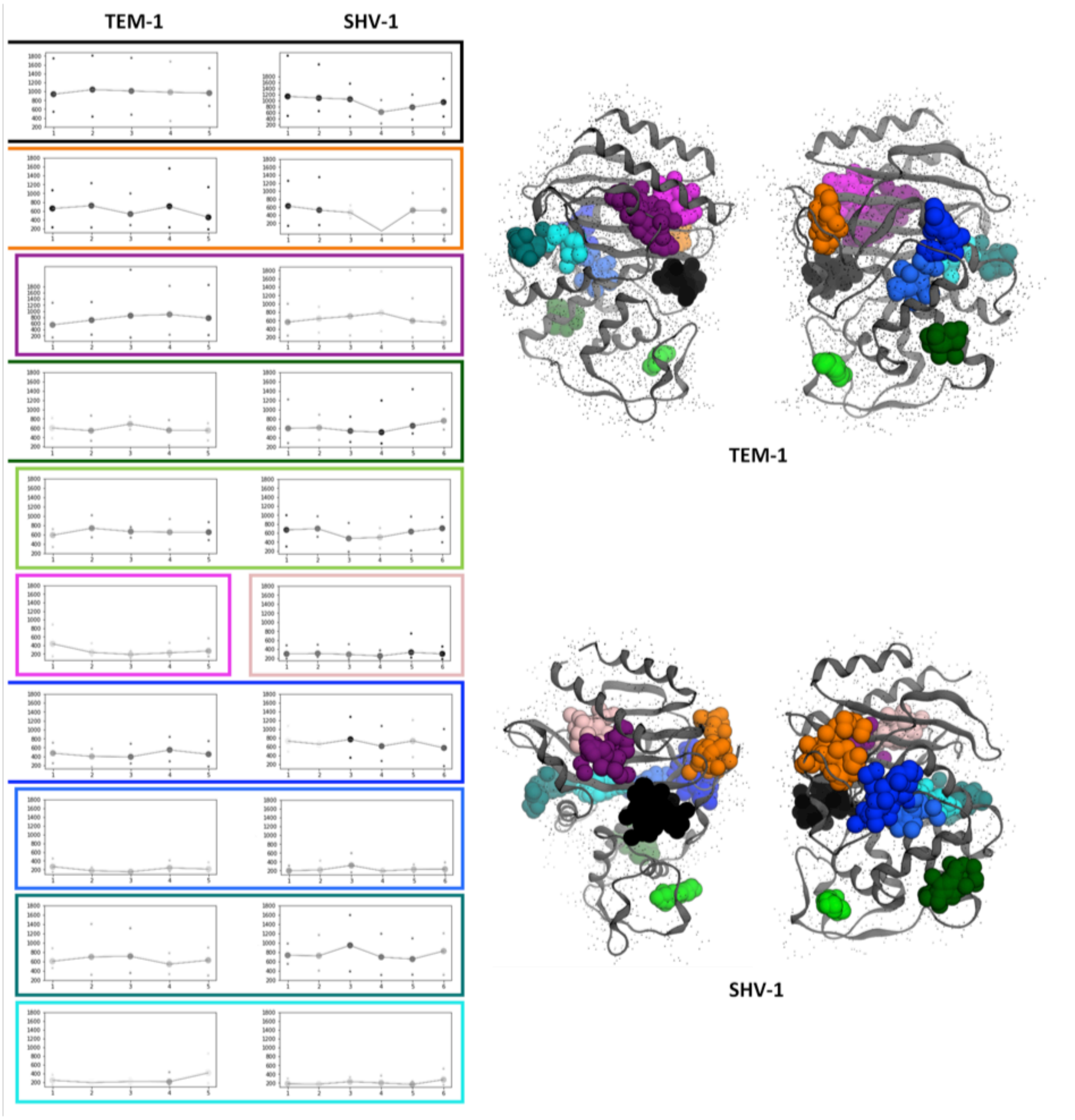

Figure S12 | TEM-1 and SHV-1 average pocket volumes (Å^3) calculated in the five metastable states. The volumes were calculated with 95% CI (based on 20 measurements or less). The number of measurements is proportional to colour intensity of the circles on the plots. The x-axis represent metastable PCCA states in which a pocket at a given location was found, and the darkness (colour intensity) of the points illustrates prevalence of the given pocket at the given metastable conformation. Pockets in cartoons are illustrated using average coordinates of fpocket vertices.

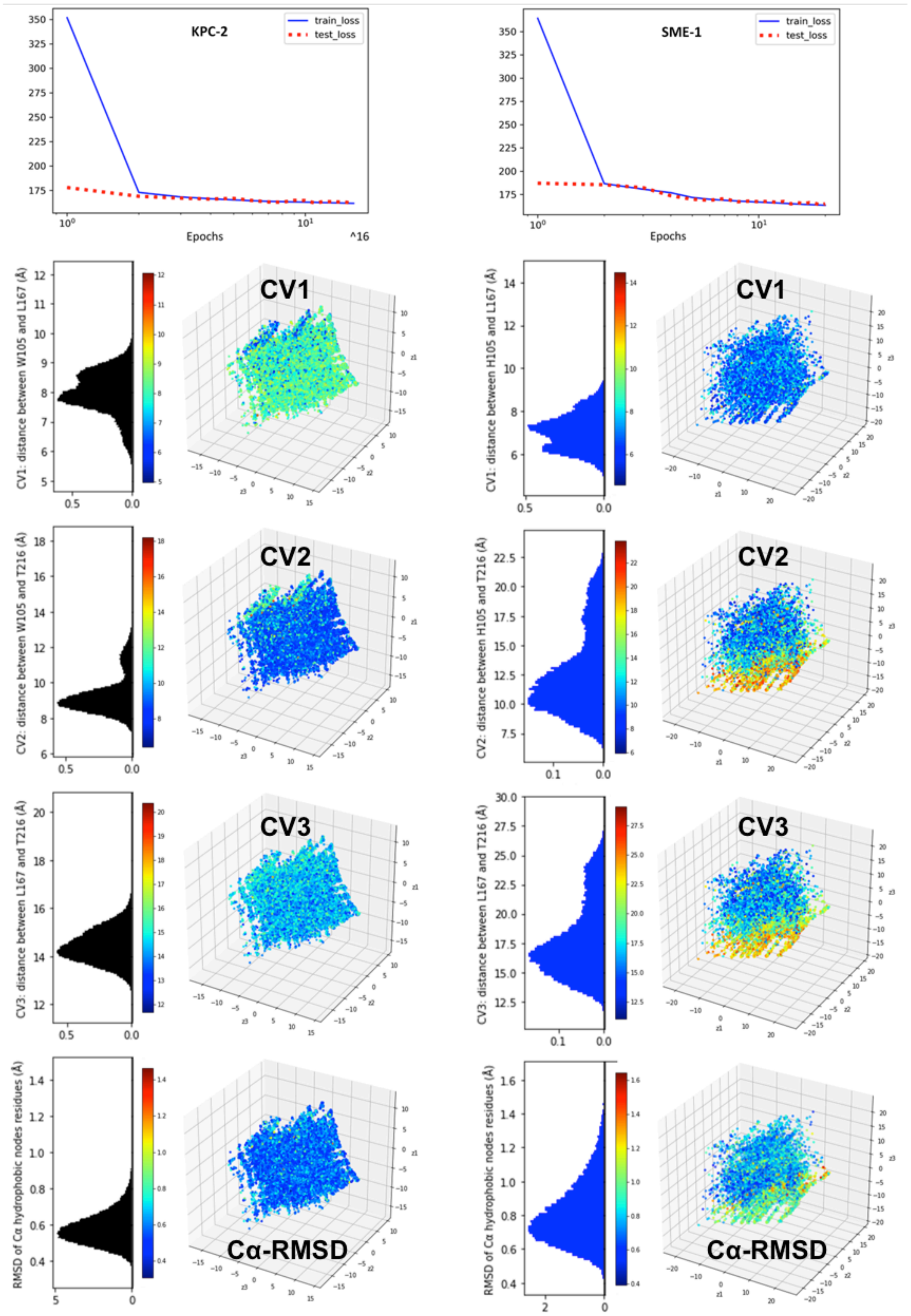

Figure S13 | KPC-2 and SME-1 CVAE latent embeddings of the hydrophobic node conformations. Dimensionality of the latent variables is three, which is visualized directly on the axes [z1, z2, z3]. CV1 represents the distance between W105 and L167; CV2 is the distance between W105 and T216 and CV3 is the distance between L167 and T216; Cα-RMSD is the RMSD of the hydrophobic node residues only.

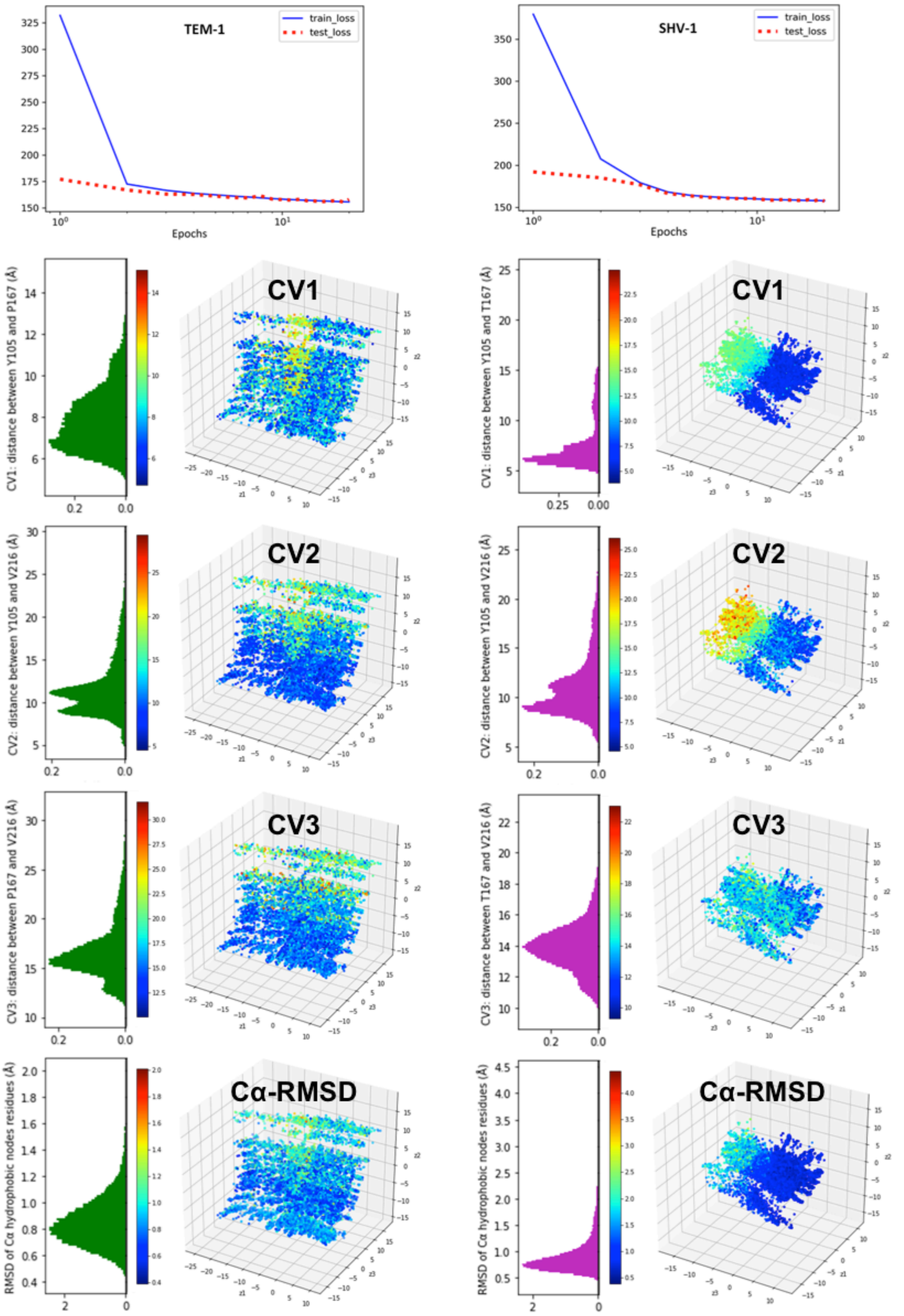

Figure S14 | TEM-1 and SHV-1 CVAE latent embeddings of the hydrophobic node conformations Dimensionality of the latent variables is three, which is visualized directly on the axes [z1, z2, z3]. CV1 represents the distance between W105 and L167; CV2 is the distance between W105 and T216 and CV3 is the distance between L167 and T216; Cα-RMSD is the RMSD of the hydrophobic node residues only.
